# The Flögel-three-component reaction with dicarboxylic acids – an approach to bis(β-alkoxy-β-ketoenamides) for the synthesis of complex pyridine and pyrimidine derivatives

**DOI:** 10.3762/bjoc.10.37

**Published:** 2014-02-13

**Authors:** Mrinal K Bera, Moisés Domínguez, Paul Hommes, Hans-Ulrich Reissig

**Affiliations:** 1 Freie Universität Berlin, Institut für Chemie und Biochemie, Takustrasse 3, D-14195 Berlin, Germany; 2 Department of Chemistry, Kamaun University, SSJ Campus Almora, Almora-263601, Uttarkhand, India

**Keywords:** alkoxyallenes, condensations, DFT calculations, β-ketoenamides, multi-component reactions, olefin metathesis, pyridines, pyrimidines

## Abstract

An extension of the substrate scope of the Flögel-three-component reaction of lithiated alkoxyallenes, nitriles and carboxylic acids is presented. The use of dicarboxylic acids allowed the preparation of symmetrical bis(β-ketoenamides) from simple starting materials in moderate yields. Cyclocondensations of these enamides to 4-hydroxypyridine derivatives or to functionalized pyrimidines efficiently provided symmetrically and unsymmetrically substituted fairly complex (hetero)aromatic compounds containing up to six conjugated aryl and hetaryl groups. In addition, subsequent functionalizations of the obtained heterocycles by palladium-catalyzed couplings or by oxidations are reported. We also describe the simple synthesis of a structurally interesting macrocyclic bispyrimidine derivative incorporating a 17-membered ring, whose configuration was elucidated by DFT calculations and by subsequent reactions.

## Introduction

**Scheme 1:**
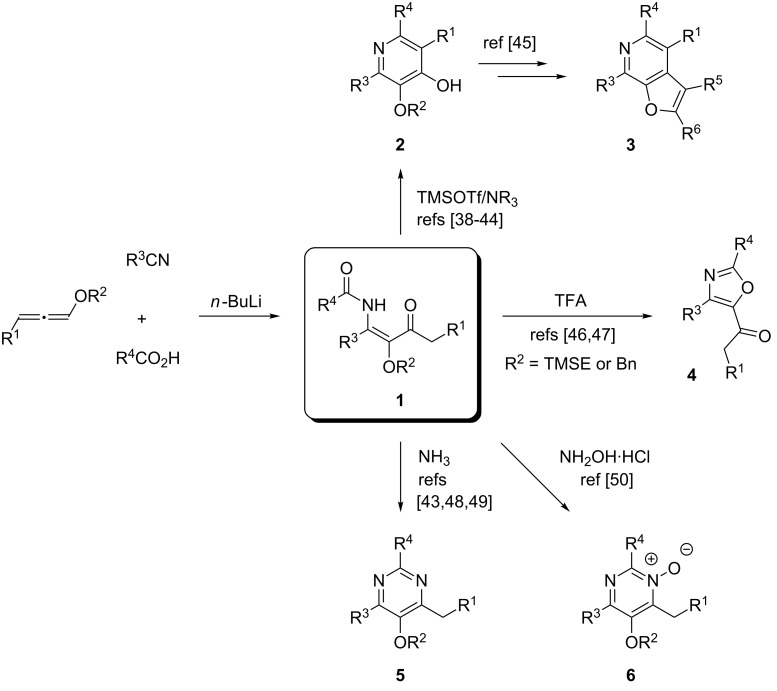
Flögel-three-component reaction of lithiated alkoxyallenes, nitriles and carboxylic acids providing β-alkoxy-β-ketoenamides **1** – versatile precursors for the synthesis of functionalized *N*-heteroaromatics **2**–**6**.

Multicomponent reactions (MCRs) generally allow a diversity-oriented fast and efficient access to complex synthetic intermediates and are thus powerful tools for the assembly of small-molecule libraries [1–2]. MCRs leading to functionalized *N*-heterocycles [3–7] have long been known before the general concept of MCRs was introduced, e.g. the Hantzsch dihydropyridine synthesis [8] or the Biginelli reaction [9] leading to dihydropyrimidinones or the corresponding dihydropyrimidinethiones. Due to their general importance (e.g. as biologically active compounds) the development of efficient protocols for the preparation of functionalized pyridine [10–20] and pyrimidine derivatives [21–33], in particular by MCRs, is of permanent high interest. In the course of exploring the reactivity of alkoxyallenes and their utilization as C-3 building blocks [34–37] our group developed a highly flexible method to synthesize β-alkoxy-β-ketoenamides of type **1** that are remarkably versatile cyclization precursors for the synthesis of functionalized heterocycles such as 4-hydroxypyridines [38–44], furopyridines [45], 5-acetyloxazoles [46–47], pyrimidines [43,48–49] and their corresponding *N*-oxides [50] (Scheme 1). This approach – discovered and mechanistically elucidated by Oliver Flögel – features a three-component reaction that employs alkoxyallenes, nitriles and carboxylic acids: upon treatment with *n*-butyllithium the allene is lithiated in α-position to the alkoxy moiety; the addition of a nitrile as electrophile to this highly reactive nucleophile results in the formation of an iminoallene adduct [38] that is protonated and subsequently acylated by the addition of a carboxylic acid furnishing a β-alkoxy-β-ketoenamide **1**. A detailed mechanistic proposal for this reaction has been disclosed in previous reports [38–39].

Our earlier investigations revealed that this method tolerates a broad variety of differently substituted starting materials – inter alia (het-)aromatic and (branched) aliphatic nitriles and carboxylic acids. It is also noteworthy to mention that the configurational integrity of enantiopure α-chiral carboxylic acids and/or nitriles is retained during this reaction [40]. In the present report we describe our efforts to further broaden the substrate scope of this multicomponent reaction and the subsequent cyclizations by employing aromatic dicarboxylic acids. This extension should allow a rapid access to fairly complex heteroaromatic systems containing up to six conjugated aryl and hetaryl groups. Complementary examples employing aromatic dinitriles in this Flögel-three-component reaction have previously been presented [39].

## Results and Discussion

**Scheme 2:**
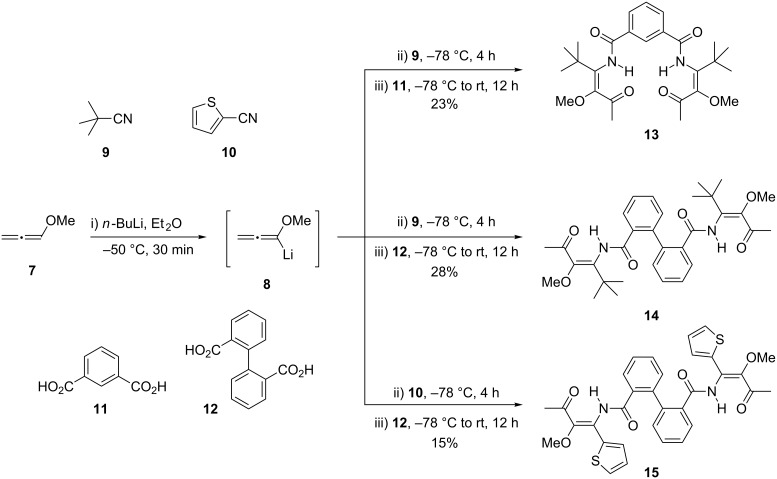
Synthesis of bis(β-ketoenamides) **13**–**15** by three-component reactions of lithiated methoxyallene **8** with nitriles **9** or **10** and isophthalic acid (**11**) or diphenic acid (**12**).

As typical model substrates we chose to employ isophthalic acid (**11**) and diphenic acid (**12**) in combination with methoxyallene (**7**), pivalonitrile (**9**) and thiophene-2-carbonitrile (**10**) in the three-component reaction (Scheme 2). Gratifyingly we were able to isolate the expected bis(β-ketoenamides) **13**–1**5** in reasonable yields of 15–28%. Taking the number of individual steps into account (six new bonds are formed for each product) and considering possible (unknown) side reactions these yields are quite satisfactory. In analogy to our previously published results [38,51–52] the double bond geometry of the enamide moiety is likely to be *E*-configured as shown in Scheme 2, allowing an intramolecular hydrogen bridge between the amide NH and the β-carbonyl group. However, we did not further investigate the nature of the double bond geometry, since it was irrelevant for the planed subsequent cyclization reactions where the (Lewis-)acidic conditions allow a facile isomerization of *E-* and *Z*-configured enamide moieties [51–52], finally leading to identical products.

**Scheme 3:**
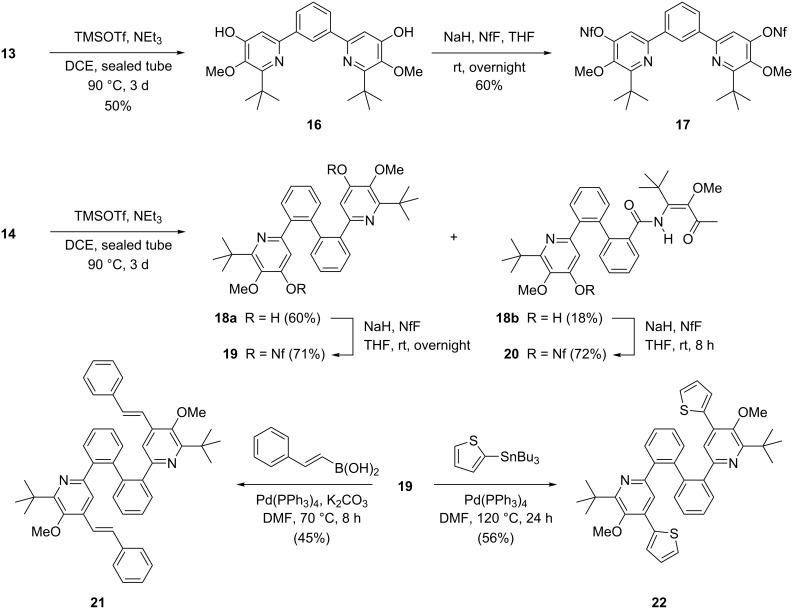
Cyclocondensations of β-ketoenamides **13** and **14** to 4-hydroxypyridines **16**, **18a** and **18b**, their subsequent nonaflations and palladium-catalyzed coupling reactions of **19** leading to compounds **21** and **22**. NfF = C_4_F_9_SO_2_F

After these successful multicomponent reactions we investigated the intramolecular condensations of the bis(β-ketoenamides) **13**–**15** to pyridine and pyrimidine derivatives. Enamides **13** and **14** were treated with trimethylsilyl trifluoromethanesulfonate (TMSOTf) and triethylamine to provide the bis(4-hydoxypyridines) **16** in 50% yield and **18a** in 60% yield, respectively (Scheme 3). A mechanistic proposal for this aldol type condensation has been presented in a previous report [53]. For precursor **14** partial monocyclization was observed under the applied conditions, affording in 18% yield 4-hydroxypyridine **18b** with a retained β-ketoenamide moiety. Treatment of compounds **16**, **18a** and **18b** with sodium hydride followed by nonafluorobutanesulfonyl fluoride (NfF) provided the corresponding sulfonates **17**, **19** and **20** in yields in the range of 60–72%. Pyrid-4-yl nonaflates are excellent precursors for transition metal-catalyzed cross-coupling reactions [42,54–58], which was demonstrated here by the successful Suzuki coupling of bisnonaflate **19** with (*E*)-styrylboronic acid and the Stille coupling of **19** with 2-(tributylstannyl)thiophene. Albeit the expected twofold coupling products **21** and **22** were obtained in only moderate yields, the presented approach nevertheless features a quite rapid access to these fairly complex heteroaromatic systems containing six conjugated aryl and hetaryl groups. Upon excitation with UV light (253 nm) compound **22** shows fluorescence with a maximum intensity at 378 nm (see Supporting Information File 1 for details). The photophysical properties of structurally related pyridine–thiophene conjugates were recently investigated in detail [55,57–58].

**Scheme 4:**
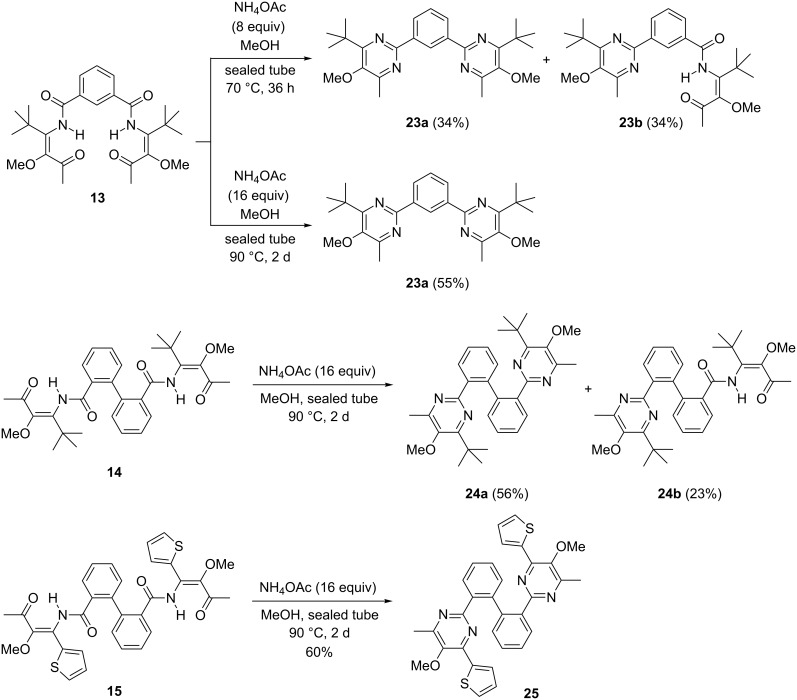
Cyclocondensations of β-ketoenamides **13**–**15** with ammonium acetate to bis(pyrimidine) derivatives **23a**, **24a** and **25** and mono-pyrimidines **23b** and **24b**.

Next, we investigated the cyclocondensation of bis(β-ketoenamides) **13**–**15** to pyrimidines (Scheme 4) using ammonium acetate as ammonia source. Initially we subjected enamide **13** to conditions that had been optimized for mono-β-ketoenamides [48–49], in this case resulting in incomplete conversion: after heating **13** with 8 equiv of ammonium acetate in a sealed tube we obtained a 1:1 mixture of bis(pyrimidine) derivative **23a** and pyrimidine **23b** still containing one β-ketoenamide unit with an overall yield of 68%. However, full conversion of **13** into **23a** was achieved by increasing the amount of ammonium acetate to 16 equiv and using a higher reaction temperature, raising the yield of **23a** from 34% to 55% yield. When enamide **14** was cyclized under these optimized conditions the conversion was nevertheless incomplete giving the desired bis(pyrimidine) derivative **24a** in 56% yield and the corresponding mono-pyrimidine **24b** in 23% yield. For enamide **15** however, the cyclization was complete under these conditions furnishing bis(pyrimidine) derivative **25** as a single product in 60% yield.

**Scheme 5:**
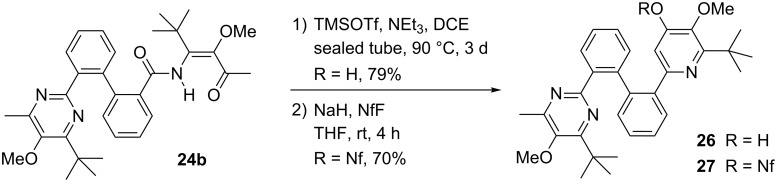
Conversion of mono-pyrimidine derivative **24b** into unsymmetrically substituted biphenylen-bridged pyrimidine/nonafloxypyridine conjugate **27**. NfF = C_4_F_9_SO_2_F

Although initially not desired the incomplete conversions of the bis(β-ketoenamides) leading to mono-pyridine derivatives such as **18b** or to mono-pyrimidine derivatives like **23b** and **24b** provided new synthetic options to construct unsymmetrically substituted mixed heteroaromatic systems. As an example we used mono-pyrimidine derivative **24b** and cyclized its β-ketoenamide moiety by treatment with TMSOTf and triethylamine. Pyrimidine/pyridinol derivative **26** was isolated in 79% yield (Scheme 5) and subsequently converted into the corresponding nonaflate **27** in 70% yield.

**Scheme 6:**
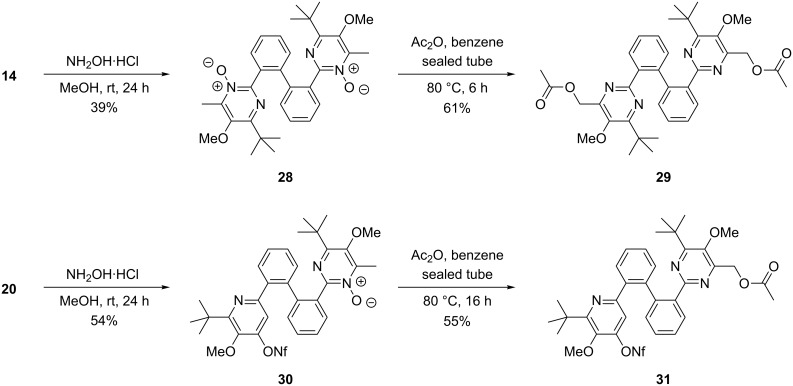
Condensation of β-ketoenamides **14** and **20** with hydroxylamine hydrochloride to pyridine-*N*-oxides **28** and **30** and their subsequent Boekelheide rearrangements furnishing functionalized bis(pyrimidine) derivative **29** and pyrimidine/pyridine conjugate **31**.

As recently described, β-alkoxy-β-ketoenamides may also be directly cyclized to pyrimidine-*N*-oxides under mild conditions if hydroxylamine hydrochloride is used as reagent [50]. Accordingly, the reactions of β-ketoenamides **14** and **20** with hydroxylamine hydrochloride provided the symmetric bis(pyrimidine-*N*-oxide) **28** in 39% yield or the mono-pyrimidine-*N*-oxide **30** in 54% yield (Scheme 6). The acetoxylation of 2- and 4-alkyl substituted pyridine-*N*-oxides by treatment with acetic anhydride is known as the Boekelheide rearrangement [59–60]. For pyrimidine-*N*-oxides however, only few examples of this type of transformation have been reported [50,61–65]. Therefore we were pleased to find that upon treatment with acetic anhydride the obtained pyrimidine-*N*-oxides **28** and **30** smoothly underwent the expected rearrangement to give the acetoxymethyl-substituted pyrimidine derivatives **29** and **31** in 61% and 55% yield, respectively. This approach thus allows the simple functionalization of the 4-methyl group of the pyrimidine derivatives and is a very useful tool for the preparation of other compounds.

**Scheme 7:**
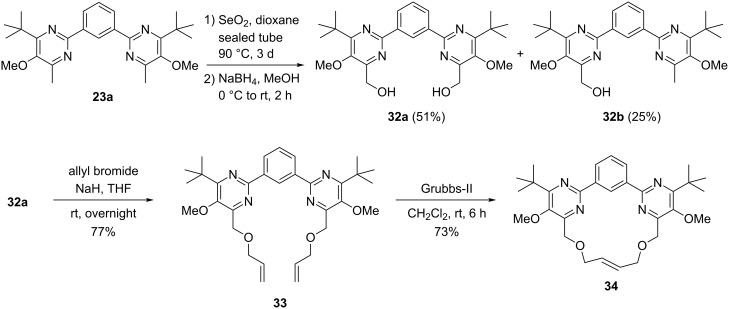
Riley oxidation of bis(pyrimidine) derivative **23a** and conversion of diol **32a** into macrocycle **34**.

An alternative option for the side chain functionalization of 4- or 6-methyl substituted pyrimidines involves an oxidation with selenium dioxide (Riley oxidation [66–68]). To explore the synthetic potential of the newly prepared compounds we exemplarily oxidized bis(pyrimidine) **23a** by this method in order to finally prepare a macrocyclic compound such as **34** (Scheme 7). Treatment of **23a** with an excess of selenium dioxide at 90 °C resulted in the formation of an inseparable mixture of two different aldehydes (probably the dialdehyde and the monoaldehyde). After reduction of the mixture with sodium borohydride the obtained products could be separated by column chromatography providing the dialcohol **32a** in 51% yield over two steps and the monoalcohol **32b** in 25% yield, respectively. The subsequent O-allylation of **32a** furnished bisallyl ether **33** with 77% yield that was subjected to a ring closing metathesis (RCM) [69] with Grubbs-II-catalyst smoothly leading to the structurally interesting macrocyclic compound **34** in 73% yield. Compounds of this type – incorporating a 17-membered ring – have the potential to serve as structurally quite unique ligands for a variety of applications, e.g. in catalysis.

**Figure 1:**
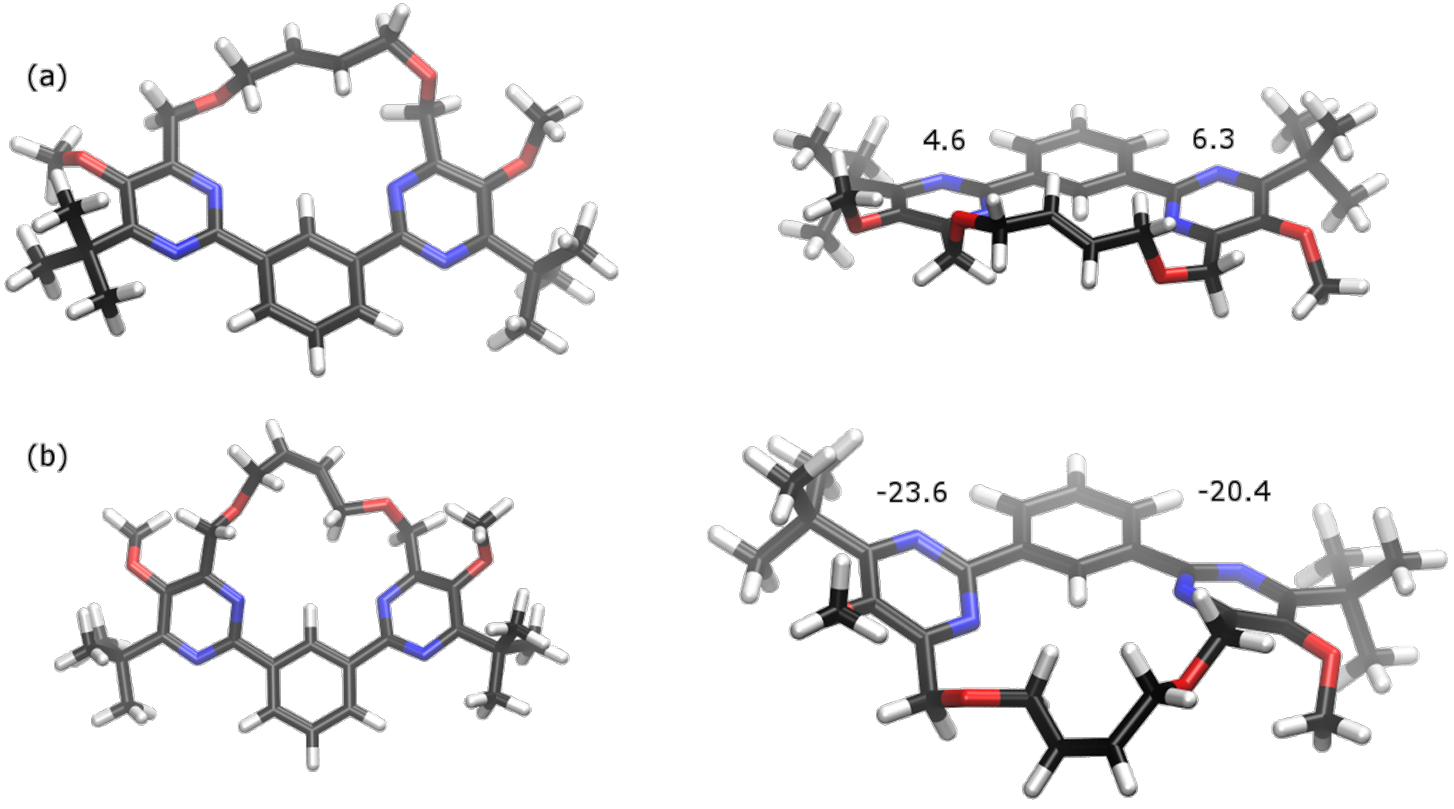
Optimized geometries of (a) *E*-configured and (b) *Z*-configured macrocycle **34** at B3LYP/6-31G(d,p) level. The numbers represent the calculated torsion angles between the aromatic rings.

With ruthenium-based catalysts bearing *N*-heterocyclic carbene (NHC) ligands, RCM usually delivers macrocyclic olefins as mixtures of *E*- and *Z*-isomers, in most cases in favor of the *E-*isomer [70–73]. The *E*/*Z*-ratio is often under thermodynamic control, reflecting the energy difference between the two isomers. According to TLC and NMR spectroscopy, macrocycle **34** was isolated as a single compound. Due to the symmetry of **34** no couplings of the olefinic protons in its ^1^H NMR spectrum can be observed. Thus at this stage, we were unable to assign the configuration of the double bond. In lack of suitable crystals for an X-ray analysis, we calculated the energy for the two possible isomers of **34**, suggesting that the *E*-isomer should be considerably more stable than the corresponding *Z*-isomer (Table 1). Using the semi-empirical AM1 method an energy difference of Δ*E_Z_*_-_*_E_* of 28.7 kJ/mol was determined. DFT calculations using the B3LYP method with the basis sets 6-31(d) or 6-31G(d,p) both gave a Δ*E_Z_*_-_*_E_* value of 16.4 kJ/mol. This energy difference may be attributed to the strain of the macrocycle and higher torsion angles between the central benzene unit and the pyrimidine rings for the *Z*-isomer of **34**, resulting in less efficient conjugation of the aromatic π-systems. The optimized molecular geometries of *E*-**34** and *Z*-**34** as well as the calculated torsion angles are depicted in Figure 1.

**Table 1:** Calculated relative energy differences of the *Z*- and *E*-configured isomers of macrocycle **34**.

Entry	Method	Δ*E_Z_*_-_*_E_* (kJ/mol)

1	AM1	28.7
2	B3LYP/6-31(d)	16.4
3	B3LYP/6-31G(d,p)	16.4

**Scheme 8:**
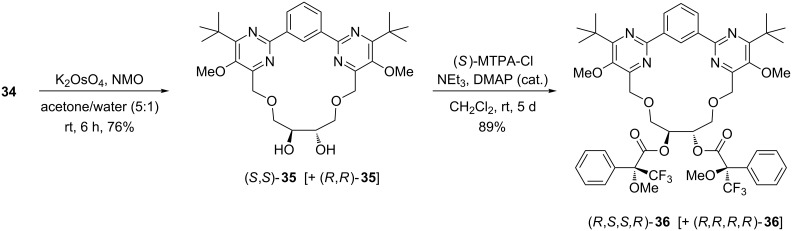
Dihydroxylation of the macrocyclic olefin **34** to diol **35** and subsequent esterification to the bis-(*R*)-Mosher ester **36**; (*S*)-MTPA-Cl = (*S*)-3,3,3-trifluoro-2-methoxy-2-phenylpropanoyl chloride.

In order to unambiguously identify the double bond configuration of **34**, we oxidized this compound with potassium osmate/NMO to obtain the vicinal diol **35** in 76% yield (Scheme 8). In the case of a *Z*-configured olefin **34** this dihydroxylation should give a *cis*-configured diol (meso compound), whereas an *E*-configured olefin **34** would lead to a racemic mixture of the corresponding *trans*-configured diol. However, due to the symmetry of both vicinal diols a distinction between *cis*- and *trans*-**35** (*σ_v_*- or *C*_2_-symmetry respectively) by NMR is still not possible. The resulting diol **35** was therefore treated with an excess of (*S*)-Mosher's acid chloride to obtain the bis-(*R*)-Mosher ester **36** [74]. TLC analysis and NMR-spectroscopy revealed, that compound **36** was obtained as a pair of *C*_2_-symmetric diastereomers and that the obtained diol **35** was in fact a racemic mixture. This observation allowed the conclusion that the RCM reaction of **33** produced the expected thermodynamically more stable *E*-configured macrocyclic olefin **34**. Hence this experimental result is in perfect agreement with the DFT calculations.

## Conclusion

We were able to extend the substrate scope of the Flögel-three-component reaction of alkoxyallenes, nitriles and carboxylic acids by successfully utilizing aromatic dicarboxylic acids to prepare three new bis(β-methoxy-β-ketoenamides). With these products of a multicomponent reaction we performed cyclizations to rapidly construct symmetrically and unsymmetrically substituted pyridine and pyrimidine derivatives. Hence a very short approach to fairly complex functionalized oligoaromatic systems was established. In addition we exemplarily investigated subsequent transformations of these compounds either by palladium-catalyzed cross-couplings or by oxidations of the 4-methyl groups of the pyrimidine subunits. Although the yields for the crucial initial multicomponent reactions leading to the bis(β-methoxy-β-ketoenamides) are only moderate when dicarboxylic acids are used the simplicity of the processes and the diversity of the products accessible is impressive. The described methods allow the preparation of oligo(hetero)aromatic compounds not available by alternative procedures.

## Experimental

### General methods

Reactions were performed under an atmosphere of argon in flame-dried flasks. Solvents and liquid reagents were added by syringe. Et_2_O, CH_2_Cl_2_ and THF were transferred from a MB SPS-800-dry solvent system into the reaction vessels. Dry DMF was purchased from Acros Organics and stored in the presence of molecular sieve under an atmosphere of argon. NEt_3_ was distilled from CaH_2_ and stored over KOH under argon. Methoxyallene was prepared from propargylic alcohol in two steps according to literature procedures [34,75]. All other solvents and reagents were purchased from commercial suppliers and were used without further purification. Thin-layer chromatography (TLC) analyses were performed on TLC plates purchased from Merck (silica gel 60, fluorescence indicator F254, 0.25 mm layer thickness). Products were purified by flash column chromatography on silica gel 60 (230–400 mesh, Macherey-Nagel). NMR spectra were recorded with Bruker (AC 500, AVIII 700) and JEOL (ECX 400, Eclipse 500) instruments. Chemical shifts are reported relative to solvent residual peaks or TMS. Integrals are in accordance with assignments, and coupling constants are given in Hz. All ^13^C NMR spectra are proton-decoupled. ^13^C NMR signals of Nf-groups [CF_3_(CF_2_)_3_] are not given since unambiguous assignment is not possible due to strong splitting by coupling with the ^19^F nuclei. IR spectra were measured with a Jasco FT/IR-4100 spectrometer. HRMS analyses were performed with a Varian Ionspec QFT-7 (ESI–FT ICRMS) or an Agilent 6210 (ESI–TOF) instrument. Melting points were measured with a Reichert apparatus (Thermovar) and are uncorrected.

#### Three-component-reaction of methoxyallene, nitriles and dicarboxylic acids (typical procedure 1)

To a solution of methoxyallene (**7**, 2.07 g, 29.6 mmol) in dry Et_2_O (25 mL) was added *n*-BuLi (10.8 mL, 27.0 mmol, 2.5 M in hexanes) at −50 °C. After 30 min stirring at −50 °C, the reaction mixture was cooled to −78 °C and pivalonitrile (**9**, 0.752 g, 9.06 mmol) in dry Et_2_O (10 mL) was added to the mixture. After stirring for 4 h a suspension of diphenic acid (**12**, 6.54 g, 27.0 mmol) in dry Et_2_O (50 mL) was added. The temperature was allowed to rise to rt and the mixture was stirred overnight. The reaction was quenched with sat. aq NaHCO_3_ solution (25 mL) and the layers were separated. The aqueous layer was extracted with Et_2_O (3 × 50 mL) and the combined organic layers were washed with brine (25 mL), dried with Na_2_SO_4_ and filtered. The solvent was removed under reduced pressure and the obtained crude product was purified by column chromatography (silica gel, hexanes/EtOAc = 1:2) to provide bis(β-ketoenamide) **14** (1.39 g, 28%) as a pale yellow solid.

***N*^2^,*N*^2'^-Bis(4-methoxy-2,2-dimethyl-5-oxohex-3-en-3-yl)biphenyl-2,2'-dicarboxamide** (**14**): mp 140–143 °C; IR (ATR) ν: 3145 (NH), 3040–2835 (=C-H, C-H), 1695 (C=O), 1525–1390 (C=C) cm^−1^; ^1^H NMR (CDCl_3_, 500 MHz) δ 0.96 (s, 18H, *t-*Bu), 2.09 (s, 6H, Me), 3.42 (s, 6H, OMe), 7.07–7.09, 7.31–7.37, 7.49–7.51 (3 m, 2H, 4H, 2H, Ar), 8.13 (br s, 2H, NH) ppm; ^13^C NMR (CDCl_3_, 126 MHz) δ 27.6 (q, Me), 28.4, 36.5 (q, s, *t-*Bu), 58.8 (q, OMe), 127.0, 127.9, 129.6, 130.4 (4 d, Ar), 131.9, 136.4, 138.4, 151.0 (4 s, C=C, Ar), 169.5 (s, CONH), 200.1 (s, C=O) ppm; ESI–TOF (*m*/*z*): [M + Na]^+^ calcd for C_32_H_40_N_2_NaO_6_, 571.2779; found, 571.2783.

#### Cyclization of β-ketoenamides to 4-hydroxypyridines (typical procedure 2)

Bis(β-ketoenamide) **14** (0.310 g, 0.57 mmol) was placed in an ACE-sealed tube and dissolved in DCE (10 mL). NEt_3_ (0.40 mL, 2.89 mmol) and TMSOTf (0.50 mL, 2.76 mmol) were added and the resulting mixture was stirred at 90 °C for 3 d. After cooling to rt the reaction was quenched with sat. aq NH_4_Cl solution (10 mL) and the layers were separated. The aqueous layer was extracted with CH_2_Cl_2_ (3 × 25 mL) and the combined organic layers were dried with Na_2_SO_4_ and filtered. The solvent was removed under reduced pressure and the obtained crude product was purified by column chromatography (silica gel, EtOAc) to provide bis(4-hydroxypyridine) **18a** (0.174 g, 60%) as a brown liquid and **18b** (54 mg, 18%) as pale yellow oil. The products were directly converted into the corresponding nonaflates **19** and **20**.

#### Nonaflation of 4-hydroxypyridines (typical procedure 3)

Bis(4-hydroxypyridine) **18a** (0.805 g, 1.57 mmol) was dissolved in THF (25 mL) and NaH (0.313 g, 7.86 mmol, 60% in mineral oil) was added under argon atmosphere. Nonafluorobutanesulfonyl fluoride (2.35 g, 7.79 mmol) was added drop-wise and the mixture was stirred at rt for 12 h. After dilution with Et_2_O (25 mL), the reaction was slowly quenched with ice and water (25 mL). The layers were separated and the aqueous layer was extracted with Et_2_O (3 × 25 mL). The combined organic layers were dried with Na_2_SO_4_, filtered and concentrated to dryness under reduced pressure. The residue was purified by column chromatography (silica gel, hexanes/EtOAc = 9:1 to 4:1) to provide pyridyl nonaflate **19** (1.20 g, 71%) as a pale yellow oil.

**6,6'-(Biphenyl-2,2'-diyl)bis(2-*tert*-butyl-3-methoxypyridine-6,4-diyl) bisnonaflate** (**19**): IR (ATR) ν: 3065–2870 (=C-H, C-H), 1555–1410 (C=C) cm^−1^; ^1^H NMR (CDCl_3_, 500 MHz) δ 1.19 (s, 18H, *t-*Bu), 3.89 (s, 6H, OMe), 6.92 (s, 2H, Py), 7.10 (dd, *J* = 7.5, 1.2 Hz, 2H, Ar), 7.30 (td, *J* = 7.5, 1.4 Hz, 2H, Ar), 7.36 (dd, *J* = 7.5, 1.4 Hz, 2H, Ar), 7.59 (dd, *J* = 7.5, 1.2 Hz, 2H, Ar) ppm; ^13^C NMR (CDCl_3_, 126 MHz) δ 29.1, 38.7 (q, s, *t-*Bu), 61.7 (q, OMe), 115.2 (d, Py), 127.4, 128.6, 130.1, 131.6 (4 d, Ar), 138.2, 140.6 (2 s, Ar), 145.3, 149.3, 153.2, 163.7 (4 s, Py) ppm; ^19^F NMR (CDCl_3_, 470 MHz) δ −80.6 (t, *J* = 9.6 Hz, 6F, CF_3_), −109.5 (t, *J* = 13.7 Hz, 4F, CF_2_), −120.7, −125.8 (2 m_c_, 4F each, CF_2_) ppm; ESI–TOF (*m*/*z*): [M + Na]^+^ calcd for C_40_H_34_F_18_N_2_NaO_8_S_2_, 1099.1361; found, 1099.1394.

#### Cyclization of β-ketoenamides to pyrimidines (typical procedure 4)

Bis(β-ketoenamide) **14** (0.162 g, 0.296 mmol) and NH_4_OAc (0.365 g, 4.73 mmol) were placed in an ACE-sealed tube. The mixture was dissolved in MeOH (5 mL) and stirred for 2 d at 90 °C. After addition of H_2_O (10 mL) and Et_2_O (20 mL) the layers were separated and the aqueous layer was extracted with Et_2_O (2 × 25 mL). The combined organic layers were dried with Na_2_SO_4_, filtered and the solvent was evaporated under reduced pressure. The residue was purified by column chromatography (silica gel, hexanes/EtOAc = 5:1) to provide pyrimidines **24a** (88 mg, 56%) and **24b** (35 mg, 23%), both as colorless oils.

**2,2'-Bis(4-*tert*-butyl-5-methoxy-6-methylpyrimidin-2-yl)biphenyl** (**24a**): IR (ATR) ν: 3070–2855 (=C-H, C-H), 1550–1440 (C=C) cm^−1^; ^1^H NMR (CDCl_3_, 500 MHz) δ 0.99 (s, 18H, *t-*Bu), 2.28 (s, 6H, Me), 3.70 (s, 6H, OMe), 7.30 (dt, *J* = 7.7, 1.9 Hz, 2H, Ar), 7.34–7.39 (m, 4H, Ar), 7.70 (dd, *J* = 7.7, 1.0 Hz, 2H, Ar) ppm; ^13^C NMR (CDCl_3_, 126 MHz) δ 19.7 (q, Me), 28.7, 37.6 (q, s, *t-*Bu), 60.9 (q, OMe), 126.4, 128.7, 130.2, 131.4 (4 d, Ar), 138.4, 142.6 (2 s, Ar), 149.8, 159.3, 159.4, 166.9 (4 s, Py) ppm; ESI–TOF (*m*/*z*): [M + H]^+^ calcd for C_32_H_39_N_4_O_2_, 511.3068; found, 511.3085.

**2'-(4-*tert*-Butyl-5-methoxy-6-methylpyrimidin-2-yl)-*N*-(4-methoxy-2,2-dimethyl-5-oxohex-3-en-3-yl)biphenyl-2-carboxamide** (**24b**): IR (ATR) ν: 3325 (N-H), 3065–2865 (=C-H, C-H), 1700, 1665 (C=O), 1550–1445 (C=C) cm^−1^; ^1^H NMR (CDCl_3_, 500 MHz) δ 0.71 (s, 9H, *t-*Bu), 1.26 (s, 9H, *t-*Bu), 2.31, 2.33 (2 s, 3H each, Me), 3.45, 3.70 (2 s, 3H each, OMe), 6.64 (dd, *J* = 7.5, 1.0 Hz, 1H, Ar), 7.07, 7.25 (2 dt, *J* = 7.5, 1.2 Hz, 1H each, Ar), 7.32 (dd, *J* = 7.5, 1.2 Hz, 1H, Ar), 7.39 (dt, *J* = 7.5, 1.8 Hz, 1H, Ar), 7.43 (dt, *J* = 7.5, 1.0 Hz, 1H, Ar), 7.50 (dd, *J* = 7.8, 1.2 Hz, 1H, Ar), 7.91 (dd, *J* = 7.8, 1.8 Hz, 1H, Ar), 8.40 (br s, 1H, NH) ppm; ^13^C NMR (CDCl_3_, 126 MHz) δ 19.2 (q, Me), 27.2 (q, Me), 28.1, 29.2, 35.9, 37.9 (2 q, 2 s, *t-*Bu), 58.9, 61.0 (2 q, OMe), 126.8, 127.9, 128.0, 128.5, 129.0, 129.4, 130.3, 130.7, 131.0 (8 d, s, Ar, =C), 137.5, 138.4, 138.9, 140.5, 150.1 (5 s, Ar, =C), 150.4, 159.6, 160.0, 168.4 (4 s, Py), 169.3 (s, CONH), 199.8 (s, C=O) ppm; ESI–TOF (*m*/*z*): [M + Na]^+^ calcd for C_32_H_34_N_3_NaO_4_, 552.2833; found, 552.2844.

## Supporting Information

File 1 Additional experimental procedures and analytical data, as well as copies of NMR spectra of representative examples.
