# Supplementary material for: The Flögel-three-component reaction with dicarboxylic acids – an approach to bis(β-alkoxy-β-ketoenamides) for the synthesis of complex pyridine and pyrimidine derivatives
Source: Beilstein J Org Chem. 2014 Feb 13;10:394–404. doi: 10.3762/bjoc.10.37 (PMC3943434; doi:10.3762/bjoc.10.37)
Supplement: File 1 — Additional experimental procedures and analytical data, as well as copies of NMR spectra of representative examples. [file Beilstein_J_Org_Chem-10-394-s001.pdf]

Supporting Information

for

The Flögel-three-component reaction with dicarboxylic acids – an approach to bis(β-alkoxy-β-ketoenamides) for the synthesis of complex pyridine and pyrimidine derivatives

Mrinal K. Bera[1,2], Moisés Domínguez[1], Paul Hommes[1] and Hans-Ulrich Reissig*,[1]

Address: [1]Freie Universität Berlin, Institut für Chemie und Biochemie, Takustrasse 3, D-14195 Berlin, Germany and [2]Current address: Department of Chemistry, Kamaun University, SSJ Campus Almora, Almora-263601, Uttarkhand, India

Email: Hans-Ulrich Reissig - hans.reissig@chemie.fu-berlin.de

*Corresponding author

Additional experimental procedures and analytical data, as well as copies of NMR spectra of representative examples

Contents:

General methods s2
Additional experimental procedures and analytical data s3
Copies of ^{1}H, ^{13}C and ^{19}F NMR spectra (representative examples) s18
UV–vis and emission spectra of compound **22** s29

General methods:

Reactions were performed under an atmosphere of argon in flame-dried flasks. Solvents and liquid reagents were added by syringe. Et_2O, CH_2Cl_2 and THF were transferred from a MB SPS-800-dry solvent system into the reaction vessels. Dry DMF was purchased from Acros Organics and stored in the presence of molecular sieve under an atmosphere of argon. NEt_3 was distilled from CaH_2 and stored over KOH under argon. Methoxyallene (**7**) was prepared from propargylic alcohol in two steps according to literature procedures [1,2]. All other solvents and reagents were purchased from commercial suppliers and were used without further purification. Thin layer chromatography (TLC) analyses were performed on TLC plates purchased from Merck (silica gel 60, fluorescence indicator F254, 0.25 mm layer thickness). Products were purified by flash column chromatography on silica gel 60 (230−400 mesh, Macherey-Nagel). NMR spectra were recorded with Bruker (AC 500, AVIII 700) and JEOL (ECX 400, Eclipse 500) instruments. Chemical shifts are reported relative to solvent residual peaks or TMS. Integrals are in accordance with assignments, and coupling constants are given in Hz. All ^{13}C NMR spectra are proton-decoupled. ^{13}C-NMR signals of Nf-groups [$CF_3(CF_2)_3$] are not given since unambiguous assignment is not possible due to strong splitting by coupling with the ^{19}F nuclei. IR spectra were measured with a Jasco FT/IR-4100 spectrometer. HRMS analyses were performed with a Varian Ionspec QFT-7 (ESI-FT ICRMS) or an Agilent 6210 (ESI-TOF) instrument. Elemental analyses were carried out with CHN-Analyzer 2400 (Perkin-Elmer), Vario EL or Vario EL III instruments. Melting points were measured with a Reichert apparatus (Thermovar) and are uncorrected. UV–vis spectra were measured with a UV–vis spectrophotometer Scinco S-3150 PDA. Fluorescence spectra were measured with a spectrofluorometer Jasco FP-6500.

[1] Hoff, S.; Brandsma, L.; Arens, J. F. *Recl. Trav. Chim. Pays-Bas* **1968**, *87*, 916–924. doi:10.1002/recl.19680870807

[2] Zimmer, R. *Synthesis* **1993**, 165–178 and references cited therein. doi:10.1055/s-1993-25823

Additional experimental procedures and analytical data

Preparation of bis(β-ketoenamide) 13:

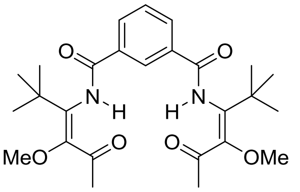

13

According to typical procedure 1, the reaction of methoxyallene (**7**) (1.49 g, 21.3 mmol), *n*-BuLi (8.00 mL, 20.0 mmol, 2.5 M in hexanes), pivalonitrile (**9**) (0.564 g, 6.78 mmol) and isophthalic acid (**11**) (3.38 g, 20.3 mmol) in dry Et_2O (50 mL) provided after stirring over night and after purification by column chromatography (silica gel, hexanes/EtOAc = 1:2) bis(β-ketoenamide) **13** (0.736 g, 23%) as a pale brown solid.

***N*^{1},*N*^{3}-Bis(4-methoxy-2,2-dimethyl-5-oxohex-3-en-3-yl)isophthalamide** (**13**): mp 125−128 °C; ^{1}H NMR ($CDCl_3$, 400 MHz): δ 1.27 (s, 18H, *t*Bu), 2.27 (s, 6H, Me), 3.55 (s, 6H, OMe), 7.48 (t, *J* = 7.8 Hz, 1H, Ar), 7.91 (dd, *J* = 7.8, 1.7 Hz, 2H, Ar), 7.94 (br s, 2H, NH), 8.21 (m_c, 1H, Ar) ppm; ^{13}C NMR ($CDCl_3$, 101 MHz): δ 27.7 (q, Me), 28.7, 36.9 (q, s, *t*Bu), 59.3 (q, OMe), 125.8, 130.6, 134.8, 135.3 (3 d, s, Ar), 150.7, 166.5 (2 s, C=C), 179.3 (s, CONH), 200.7 (s, C=O) ppm; ESI–TOF (*m*/*z*): $[M + Na]^+$ calcd for $C_{26}H_{36}N_2NaO_6$, 495.2466; found, 495.2483.

Preparation of bis(β-ketoenamide) 15:

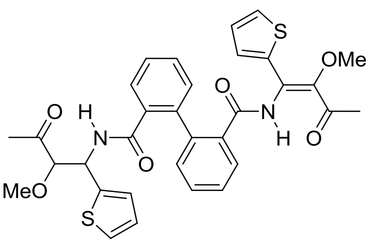

15

According to typical procedure 1, the reaction of methoxyallene (**7**) (1.25 g, 17.8 mmol), *n*-BuLi (6.50 mL, 16.3 mmol, 2.5 M in hexanes), thiophene-2-carbonitrile (**10**) (0.586 g, 5.37 mmol) and diphenic acid (**12**) (3.90 g, 16.1 mmol) in dry Et_2O (50 mL) provided after stirring over night and after purification by column chromatography (silica gel, hexanes/EtOAc = 1:1) bis(β-ketoenamide) **15** (0.484 g, 15%) as a brownish foam.

***N*^{2},*N*$^{2'}$-Bis[2-methoxy-3-oxo-1-(2-thiophenyl)but-1-enyl]biphenyl-2,2'-dicarboxamide** (**15**): IR (ATR) ν: 3330 (NH), 3005–2935 (=C-H, C-H), 1650 (C=O), 1575–1420 (C=C) cm^{-1}; ^{1}H NMR ($CDCl_3$, 400 MHz): δ 2.27 (s, 6H, Me), 3.56 (s, 6H, OMe), 6.49–6.51, 6.74–6.76 (2 m, 2H each, Thio), 7.30–7.35, 7.48–7.51 (2 m, 4H each,

Ar), 7.77–7.79 (m, 2H, Thio), 9.78 (s, 2H, NH) ppm; ^{13}C NMR ($CDCl_3$, 101 MHz): δ 28.0 (q, Me), 59.8 (q, OMe), 126.4, 127.8, 128.3, 129.4, 130.1, 130.2, 130.5, 130.6, 134.1, 136.2 (7 d, 3 s, Thio, Ar), 138.7, 144.2 (2 s, C=C), 169.6 (s, CONH), 198.6 (s, C=O) ppm; ESI–TOF (*m*/*z*): $[M + Na]^+$ calcd for $C_{32}H_{28}N_2NaO_6S_2$, 623.1281; found, 623.1302.

Cyclization of 13 to bispyridinol 16:

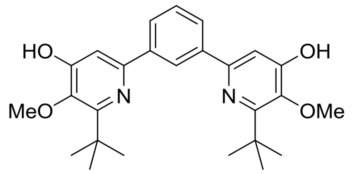

16

According to typical procedure 2, the reaction of enamide **13** (0.300 g, 0.64 mmol), NEt_3 (0.50 mL, 3.59 mmol) and TMSOTf (0.60 mL, 3.32 mmol) in DCE (10 mL) provided after purification by column chromatography (silica gel, EtOAc) pyridinol **16** (0.138 g, 50%) as a brownish foam.

6,6'-(1,3-phenylene)bis(2-*tert*-butyl-3-methoxypyridin-4-ol) (**16**): IR (ATR) ν: 3675–3435 (NH/OH), 2955–2880 (C-H), 1580–1525 (C=C) cm^{-1}; ^{1}H NMR (CD_3OD, 400 MHz): δ 1.47 (s, 18H, *t*Bu), 3.88 (s, 6H, OMe), 6.93 (br s, 2H, Py), 7.56 (t, *J* = 7.7 Hz, 1H, Ar), 7.81 (br s, 2H, Ar), 8.23 (br s, 1H, Ar) ppm, large signal broadening due to pyridinol/pyridine tautomerism was observed; a ^{13}C NMR spectrum with satisfactory resolution could not be obtained; ESI–TOF (*m*/*z*): $[M + H]^+$ calcd for $C_{26}H_{33}N_2O_4$, 437.2435; found, 437.2417.

Nonaflation of 16:

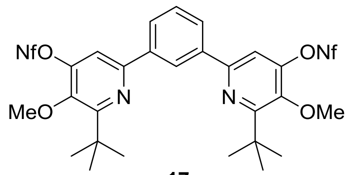

17

According to typical procedure 3, the reaction of pyridinol **16** (92 mg, 0.21 mmol), NaH (42 mg, 1.05 mmol, 60% in mineral oil), and NfF (336 mg, 1.11 mmol) in THF (5 mL) provided after stirring over night at rt and after purification by column chromatography (silica gel, hexanes/EtOAc = 9:1 to 4:1) bisnonaflate **17** (126 mg, 60%) as a pale yellow oil.

6,6'-(1,3-Phenylene)bis(2-*tert*-butyl-3-methoxypyridine-6,4-diyl) bisnonaflate (**17**): IR (ATR) ν: 3080, 2960–2870 (=C-H, C-H), 1555–1405 (C=C) cm^{-1}; ^{1}H NMR ($CDCl_3$, 500 MHz): δ 1.50 (s, 18H, *t*Bu), 3.97 (s, 6H, OMe), 7.56 (t, *J* = 7.7 Hz, 1H, Ar), 7.58 (s, 2H, Py), 8.01 (dd, *J* = 7.7, 1.7 Hz, 2H, Ar), 8.77 (t, *J* = 1.7 Hz, 1H, Ar) ppm; ^{13}C NMR

(CDCl$_3$, 126 MHz): δ 29.3, 39.2 (q, s, *t*Bu), 61.9 (q, OMe), 111.3 (d, Py), 125.2, 127.3, 129.3, 138.4 (3 d, s, Ph), 146.3, 150.5, 151.0, 164.4 (4 s, Py) ppm; ^{19}F NMR (CDCl$_3$, 470 MHz): δ –80.5 (t, *J* = 9.7 Hz, 6F, CF$_3$), –109.4 (t, *J* = 13.7 Hz, 4F, CF$_2$), –120.6, –125.7 (2 m$_c$, 4F each, CF$_2$) ppm; ESI–TOF (*m*/*z*): [M + H]$^+$ calcd for $C_{34}H_{31}F_{18}N_2O_8S_2$, 1001.1229; found, 1001.1279.

Nonaflation of 18b:

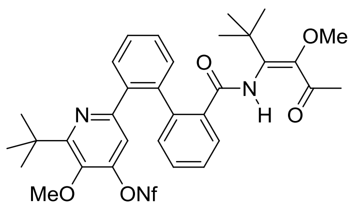

20

According to typical procedure 3, the reaction of pyridinol **18b** (123 mg, 0.232 mmol), NaH (47 mg, 1.18 mmol, 60% in mineral oil), and NfF (336 mg, 1.11 mmol) in dry THF (5 mL) provided after stirring over night at rt and after purification by column chromatography (silica gel, hexanes/EtOAc = 9:1 to 3:1) pyridyl nonaflate **20** (135 mg, 72%) as a pale yellow oil.

2-*tert*-Butyl-3-methoxy-6-[2'-(4-methoxy-2,2-dimethyl-5-oxohex-3-en-3-ylcarbamoyl) biphenyl-2-yl]pyridin-4-yl nonaflate (**20**): ^{1}H NMR (CDCl$_3$, 500 MHz): δ 0.79, 1.10 (2 s, 9H each, *t*Bu), 2.26 (s, 3H, Me), 3.44, 3.86 (2 s, 3H each, OMe), 6.60 (br d, *J* = 7.5 Hz, 1H, Ar), 7.07 (dt, *J* = 7.5, 1.0 Hz, 1H, Ar), 7.21 (br s, 1H, NH), 7.22−7.25 (m, 1H, Ar), 7.28 (s, 1H, Py), 7.33 (dd, *J* = 7.2, 1.6 Hz, 1H, Ar), 7.37−7.43 (m, 2H, Ar), 7.50 (br d, *J* = 7.2 Hz, 1H, Ar), 7.59 (dd, *J* = 7.5, 1.6 Hz, 1H, Ar) ppm; ^{13}C NMR (CDCl$_3$, 126 MHz): δ 27.0 (q, Me), 28.2, 29.3, 35.9, 38.6 (2 s, 2 q, *t*Bu), 59.0, 61.8 (2 q, OMe), 115.9 (d, Py), 127.0, 128.0, 128.3, 129.0, 129.1, 129.9, 130.2, 130.5 (8 d, Ar), 131.5, 137.2, 138.4, 138.5, 139.6, 145.7, 149.7, 150.2, 153.7, 164.4 (10 s, C=C, Py, Ar), 168.7 (s, CONH), 199.7 (s, C=O) ppm; ^{19}F NMR (CDCl$_3$, 470 MHz): δ −80.6 (t, *J* = 9.6 Hz, 3F, CF$_3$), −109.5, −120.7, −125.7 (3 m$_c$, 2F each, CF$_2$) ppm.

Suzuki coupling with 19:

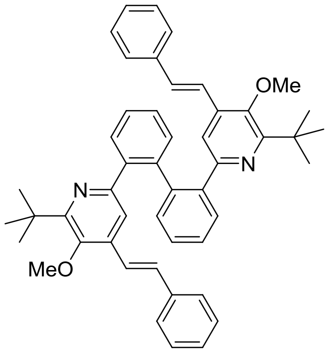

21

A mixture of bisnonaflate **19** (100 mg, 0.093 mmol), $Pd(PPh_3)_4$ (21 mg, 0.018 mmol), K_2CO_3 (128 mg, 0.93 mmol) and (*E*)-styrylboronic acid (137 mg, 0.93 mmol) in DMF (5 mL) was heated to 70 °C for 8 h under an argon atmosphere. The mixture was cooled to room temperature, diluted with brine (5 mL) and extracted with Et_2O (3 × 15 mL). The combined organic phases were dried with Na_2SO_4, filtered and concentrated to dryness. The residue was purified by column chromatography (silica gel, hexanes/EtOAc = 19:1 to 9:1) to give compound **21** (28 mg, 45%) as a pale yellow oil.

2,2'-Bis(6-*tert*-butyl-5-methoxy-4-styrylpyridin-2-yl)biphenyl (**21**): UV–vis ($CHCl_3$, log ε) λ_{max}: 293 nm (4.57); ^1H NMR ($CDCl_3$, 500 MHz): δ 1.11 (s, 18H, *t*Bu), 3.58 (s, 6H, OMe), 6.69 (d, *J* = 16.4 Hz, 2H, =CH), 6.73 (s, 2H, Py), 7.04 (d, *J* = 16.4 Hz, 2H, =CH), 7.29−7.36, 7.38−7.40 (2 m, 6H each, Ph, Ar), 7.42−7.46, 7.47−7.52 (2 m, 3H each, Ph, Ar) ppm; ^{13}C NMR ($CDCl_3$, 126 MHz): δ 29.5, 37.9 (s, q, *t*Bu), 62.5 (q, OMe), 118.4, 122.9, 126.4, 126.8, 126.9, 128.2, 128.7, 128.9, 129.3, 129.6 (10 d, CH=CH, Ph, Ar, Py), 131.9, 136.9, 139.7, 141.9, 151.3, 151.7, 159.9 (7 s, Ph, Ar, Py) ppm; ESI–TOF (*m*/*z*): $[M + H]^+$ calcd for $C_{48}H_{49}N_2O_2$, 685.3889; found, 685.3807.

Stille-coupling with 19:

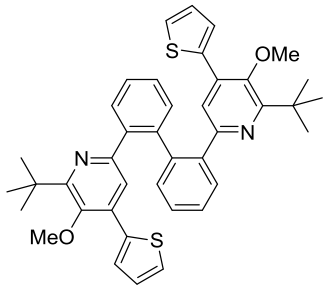

22

A mixture of bisnonaflate **19** (110 mg, 0.102 mmol), 2-(tributylstannyl)thiophene (0.352 g, 0.945 mmol), and $Pd(PPh_3)_4$ (12 mg, 0.010 mmol) in dry DMF (4 mL) was stirred at 120 °C for 24 h under an argon atmosphere. The mixture was diluted with Et_2O (10 mL) and water (10 mL). The layers were separated and the aquoeus layer was extracted with Et_2O (3 × 25 mL). The combined organic layers were dried with Na_2SO_4, filtered and concentrated to dryness. The residue was purified by column chromatography (silica gel, hexanes/EtOAc = 9:1 to 3:1) to provide compound **22** (37 mg, 56%) as a colorless oil.

2,2'-Bis[6-*tert*-butyl-5-methoxy-4-(2-thiophenyl)pyridin-2-yl]biphenyl (**22**): IR (ATR) ν: 3000–2815 (=C-H, C-H), 1540–1405 (C=C) cm^{-1}; UV–vis (MeCN, log ε) λ_{max}: 253 nm (4.51); PL (MeCN, excitation at 253 nm) λ_{max}: 378 nm; ^{1}H NMR ($CDCl_3$, 500 MHz): δ 1.13 (s, 18H, *t*Bu), 3.24 (s, 6H, OMe), 6.86 (s, 2H, Py), 6.98 (dd, *J* = 5.1, 3.5 Hz, 2H, Thio), 7.16 (dd, *J* = 3.5, 1.1 Hz, 2H, Thio), 7.30 (dd, *J* = 5.1, 1.1 Hz, 2H, Thio), 7.34–7.39, 7.54–7.55 (2 m, 6H, 2H, Ar) ppm; ^{13}C NMR ($CDCl_3$, 126 MHz): δ 29.7, 38.1 (q, s, *t*Bu), 60.5 (q, OMe), 121.8 (d, Py), 127.0,* 127.2, 127.5, 127.8, 128.3, 129.8, 132.0 (7 d, Thio, Ar, Py), 134.8, 138.0, 139.2, 141.7, 150.3, 151.9, 160.7 (7 s, Thio, Ar, Py) ppm, *intensity of the peak corresponds to two C atoms; ESI–TOF (*m*/*z*): $[M + H]^+$ calcd for $C_{40}H_{41}N_2O_2S_2$, 645.2604; found, 645.2585.

Cyclization of 13 to pyrimidines:

Conditions A: Analogously to typical procedure 4, the reaction of enamide **13** (0.224 g, 0.475 mmol) and NH_4OAc (0.293 g, 3.80 mmol) in MeOH (5 mL) at 70 °C for 36 h provided after purification by column chromatography (silica gel, hexanes/EtOAc = 9:1 to 3:1) pyrimidines **23a** (70 mg, 34%) and **23b** (73 mg, 34%), both as a colorless oils.

Conditions B: According to typical procedure 4, the reaction of enamide **13** (0.155 g, 0.328 mmol) and NH_4OAc (0.405 g, 5.25 mmol) in MeOH (5 mL) at 90 °C for 2 d provided after purification by column chromatography (silica gel, hexanes/EtOAc = 9:1 to 3:1) bispyrimidine **23a** (78 mg, 55%) as single product.

1,3-Bis(4-*tert*-butyl-5-methoxy-6-methylpyrimidin-2-yl)benzene (**23a**):

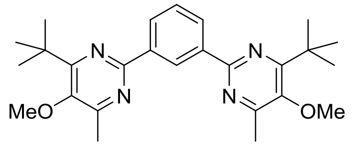

23a

IR (ATR) ν: 3030–2865 (=C-H, C-H), 1550–1375 (C=C) cm^{-1}; ^{1}H NMR ($CDCl_3$, 400 MHz): δ 1.49 (s, 18H, *t*Bu), 2.59 (s, 6H, Me), 3.81 (s, 6H, OMe), 7.53 (t, *J* = 7.9 Hz, 1H, Ar), 8.47 (dd, *J* = 7.9, 1.6 Hz, 2H, Ar), 9.53 (t, *J* = 1.6 Hz, 1H, Ar) ppm; ^{13}C NMR ($CDCl_3$, 101 MHz): δ 20.0 (q, Me), 29.2, 38.3 (q, s, *t*Bu), 61.1 (q, OMe), 128.0, 128.6, 129.2, 138.4 (3 d, s, Ar), 151.0, 157.5, 160.4, 167.9 (4 s, Pyr) ppm; ESI–TOF (*m*/*z*): $[M + H]^+$ calcd for $C_{26}H_{35}N_4O_2$, 435.2755; found, 435.2753.

3-(4-*tert*-Butyl-5-methoxy-6-methylpyrimidin-2-yl)-*N*-(4-methoxy-2,2-dimethyl-5-oxohex-3-en-3-yl)benzamide (**23b**):

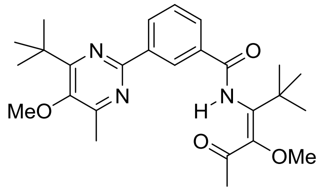

23b

IR (ATR) ν: 3325 (NH), 3060–2865 (=C-H, C-H), 1695, 1665 (C=O), 1550–1445 (C=C) cm^{-1}; ^{1}H NMR ($CDCl_3$, 500 MHz): δ 1.33, 1.47 (2 s, 9H each, *t*Bu), 2.32, 2.57 (2 s, 3H each, Me), 3.58, 3.81 (2 s, 3H each, OMe), 7.52 (t, *J* = 7.8 Hz, 1H, Ar), 7.97 (br s, 1H, Ar), 7.88, 8.58 (2 br d, *J* = 7.8 Hz, 1H each, Ar), 8.83 (br s, 1H, NH) ppm; ^{13}C NMR ($CDCl_3$, 126 MHz): δ 19.9, 27.6 (2 q, Me), 28.6, 29.2, 36.9, 38.2 (2 q, 2 s, *t*Bu), 59.1, 61.0 (2 q, OMe), 126.2, 128.7, 128.9, 131.3, 134.5, 134.6, 138.6, 150.5 (4 d, 4 s, Ar, C=C), 151.3, 156.4, 160.6, 167.2 (4 s, Pyr), 168.1 (s, CONH), 200.7 (s, C=O) ppm; ESI–TOF (*m*/*z*): [M + Na]$^+$ calcd for $C_{26}H_{35}N_3NaO_4$, 476.2520; found, 476.2517.

Cyclization of 15 to pyrimidine 25:

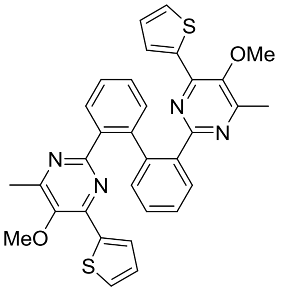

25

According to typical procedure 4, the reaction of enamide **15** (90 mg, 0.15 mmol) and NH_4OAc (0.185 g, 2.40 mmol) in MeOH (2 mL) at 90 °C for 2 d provided after purification by column chromatography (silica gel, hexanes/EtOAc = 9:1 to 4:1) bispyrimidine **25** (50 mg, 60%) as a pale yellow solid.

1,3-Bis[5-methoxy-4-methyl-6-(2-thiophenyl)pyrimidin-2-yl]-benzene (**25**): mp 137−140 °C; IR (ATR) ν: 3100–2850 (=C-H, C-H), 1550, 1430 (C=C) cm^{-1}; ^{1}H NMR ($CDCl_3$, 400 MHz): δ 2.15 (s, 6H, Me), 3.60 (s, 6H, OMe), 7.00 (dd, *J* = 5.0, 3.7 Hz, 2H, Thio), 7.34 (dt, *J* = 7.4, 1.4 Hz, 2H, Ar), 7.39 (dd, *J* = 5.0, 1.2 Hz, 2H, Thio), 7.54 (dt, *J* = 7.4, 1.4 Hz, 2H, Ar), 7.57 (dd, *J* = 3.7, 1.2 Hz, 2H, Thio), 7.62 (dd, *J* = 7.6, 1.0 Hz, 2H, Ar), 7.70 (dd, *J* = 7.6, 1.0 Hz, 2H, Ar) ppm; ^{13}C NMR ($CDCl_3$, 101 MHz): δ 18.9 (q, Me), 59.9 (q, OMe), 126.7, 127.6, 129.3, 129.8, 130.2, 130.2, 131.0 (7 d, Ar, Thio), 138.0, 138.7, 142.4 (3 s, Ar, Thio), 145.8, 150.3, 160.5, 161.0 (4 s, Py) ppm; ESI–TOF (*m*/*z*): [M + H]$^+$ calcd for $C_{32}H_{27}N_4O_2S_2$, 563.1570; found, 563.1606.

Cyclization 24b to 4-hydroxypyridine 26:

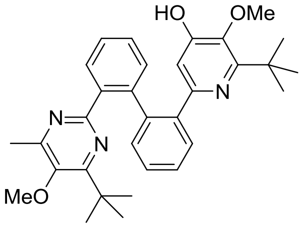

According to typical procedure 2, the reaction of enamide **24b** (85 mg, 0.16 mmol), NEt_3 (0.10 mL, 0.72 mmol), and TMSOTf (0.15 mL, 0.83 mmol) in DCE (5 mL) afforded after purification by column chromatography (silica gel, EtOAc) compound **26** (65 mg, 79%) as a brownish oil.

2-*tert*-Butyl-6-[2'-(4-*tert*-butyl-5-methoxy-6-methyl-pyrimidin-2-yl)biphenyl-2-yl]-3-methoxypyridin-4-ol (**26**): IR (ATR) ν: 3500 (OH), 3065–2870 (=C-H, C-H), 1620–1480 (C=C) cm^{-1}; ^1H NMR (CD_3OD, 400 MHz): δ 0.96, 1.08 (2 s, 9H each, *t*Bu), 2.39 (s, 3H, Me), 3.77, 3.83 (2 s, 3H each, OMe), 6.55 (s, 1H, Py), 6.84 (dd, *J* = 7.7, 1.0 Hz, 1H, Ar), 7.08 (dd, *J* = 7.6, 1.0 Hz, 1H, Ar), 7.29 (dt, *J* = 7.6, 1.2 Hz, 1H, Ar), 7.38–7.41 (m, 2H, Ar), 7.49 (dt, *J* = 7.7, 1.2 Hz, 1H, Ar), 7.55, 7.92 (2 dd, *J* = 7.6, 1.0 Hz, 1H, Ar) ppm; ^{13}C NMR (CD_3OD, 101 MHz): δ 18.5 (q, Me), 27.3, 28.1, 35.2, 37.5 (2 q, 2 s, *t*Bu), 58.4, 60.6 (2 q, OMe), 116.4, 118.8, 122.0, 127.6, 127.9, 129.2, 129.4, 129.6, 130.1, 130.3, 130.4, 132.7, 138.7, 139.7, 140.9, 146.6, 148.9 (9 d, 8 s, Ar, Py), 150.5, 158.9, 160.7, 168.0 (4 s, Pyr) ppm; ESI–TOF (*m*/*z*): $[M + H]^+$ calcd for $C_{32}H_{38}N_3O_3$, 512.2908; found, 512.2913.

Nonaflation of 26:

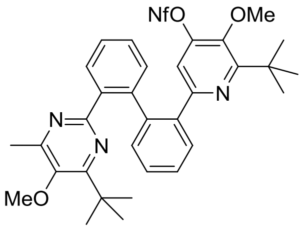

According to typical procedure 3, the reaction of pyridinol **26** (65 mg, 0.13 mmol), NaH (25 mg, 0.63 mmol, 60% in mineral oil), and NfF (168 mg, 0.56 mmol) in THF (5 mL) provided after stirring over night at rt and after purification by column chromatography (silica gel, hexanes/EtOAc = 9:1 to 3:1) nonaflate **27** (71 mg, 70%) as a pale yellow oil.

2-*tert*-Butyl-6-[2'-(4-*tert*-butyl-5-methoxy-6-methylpyrimidin-2-yl)biphenyl-2-yl]-3-methoxypyridin-4-yl nonaflate (**27**): IR (ATR) ν: 3060–2870 (=C-H, C-H), 1550–1380 (C=C) cm^{-1}; ^1H NMR ($CDCl_3$, 400 MHz): δ 0.98, 1.37 (2 s, 9H each, *t*Bu), 2.44 (s, 3H, Me), 3.74, 3.87 (2 s, 3H each, OMe), 6.97 (dd, *J* = 6.2, 0.5 Hz, 1H, Ar), 7.01 (s, 1H, Py), 7.02 (dd, *J* = 6.2, 0.5 Hz, 1H, Ar), 7.20 (dd, *J* = 6.1, 1.0 Hz, 1H, Ar), 7.25 (dd, *J* = 7.1, 1.2 Hz, 1H, Ar), 7.30–7.36 (m, 2H, Ar), 7.72 (dd, *J* = 6.1, 1.0 Hz, 1H, Ar), 7.88 (br d, *J* =

6.1 Hz, 1H, Ar) ppm; ^{13}C NMR ($CDCl_3$, 101 MHz): δ 19.8 (q, Me), 28.8, 29.5, 37.7, 38.8 (2 q, 2 s, *t*Bu), 60.9, 61.7 (2 q, OMe), 116.0 (d, Py), 126.7, 127.3, 128.0, 128.7, 130.0, 130.6, 130.7, 131.7 (8 d, Ar), 138.2, 138.5, 140.9, 141.9, 145.2, 149.1, 150.1, 153.4 (8 s, Ar, Py), 159.6, 160.0, 163.3, 167.4 (4 s, Py) ppm; ESI–TOF (*m*/*z*): $[M + H]^+$ calcd for $C_{36}H_{37}F_9N_3O_5S$, 794.2305; found, 794.2338.

Cyclization of to pyrimidine-*N*-oxides (typical procedure 5):

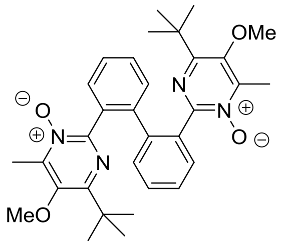

28

Bis(β-ketoenamide) **14** (110 mg, 0.20 mmol) was dissolved in MeOH (5 mL) and NH_2OH•HCl (0.280 g, 4.01 mmol) was added. The solution was stirred at rt for 1 d. After addition of H_2O (10 mL), the mixture was extracted with EtOAc (3 × 25 mL). The combined organic layers were dried with Na_2SO_4, filtered and concentrated. Column chromatography (silica gel, EtOAc) provided bis(*N*-oxide) **28** (42 mg, 39%) as a colorless viscous oil.

2,2'-(Biphenyl-2,2'-diyl)bis(4-*tert*-butyl-5-methoxy-6-methylpyrimidine 1-oxide) (**28**): ^{1}H NMR ($CDCl_3$, 500 MHz): δ 0.99 (s, 18H, *t*Bu), 2.38 (s, 6H, Me), 3.84 (s, 6H, OMe), 7.28 (dt, *J* = 7.8, 1.2 Hz, 2H, Ar), 7.38–7.51 (m, 4H, Ar), 7.77 (br d, *J* = 7.8 Hz, 2H, Ar) ppm; ^{13}C NMR ($CDCl_3$, 126 MHz): δ 12.5 (q, Me), 28.8, 37.5 (q, s, *t*Bu), 61.7 (q, OMe), 125.8, 129.3, 129.5, 131.4, 132.6 (3 d, 2 s, Ar), 142.0, 150.3, 150.4, 155.8 (4 s, Py) ppm. The signal for one aromatic C atom could not be detected.

Boekelheide-rearrangement of methylpyrimidine-*N*-oxides (typical procedure 6):

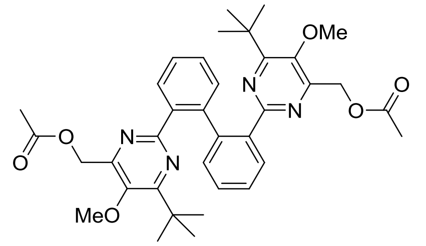

29

A solution of bis(pyrimidine-*N*-oxide) **28** (40 mg, 0.074 mmol) and Ac_2O (0.10 mL, 1.1 mmol) in benzene (2 mL) was heated to 80 °C for 6 h in an ACE sealed tube. After cooling to rt, the mixture was diluted with water (10 mL) and Et_2O (20 mL). The layers were separated and the organic layer was washed with brine (15 mL), dried with Na_2SO_4 and filtered. The solvents were evaporated under reduced pressure and the remaining residue was purified by column

chromatography (silica gel, hexanes/EtOAc = 4:1) to afford compound **29** (27 mg, 61%) as a colorless oil.

2,2'-(Biphenyl-2,2'-diyl)bis(6-*tert*-butyl-5-methoxypyrimidine-4,2-diyl)bis(methylene) diacetate (**29**): IR (ATR) ν: 3060−2865 (=C-H, C-H), 1740 (C=O), 1555−1380 (C=C) cm^{-1}; ^{1}H NMR ($CDCl_3$, 500 MHz): δ 0.98 (s, 18H, *t*Bu), 2.14 (s, 6H, Me), 3.76 (s, 6H, OMe), 5.02, 5.10 (AB system, J_{AB} = 13.1 Hz, 4H, CH_2OAc), 7.27−7.32 (m, 4H, Ar), 7.34 (dd, *J* = 7.4, 1.2 Hz, 2H, Ar), 7.74 (dd, *J* = 7.4, 1.2 Hz, 2H, Ar) ppm; ^{13}C NMR ($CDCl_3$, 126 MHz): δ 20.9 (q, Me), 28.8, 37.9 (q, s, *t*Bu), 62.1 (t, OCH_2), 62.5 (q, OMe), 126.5, 128.9, 130.5, 131.4 (4 d, Ar), 137.9, 142.6 (2 s, Ar), 149.6, 155.8, 159.8, 168.5 (4 s, Pyr), 170.8 (s, C=O) ppm; ESI–TOF (*m*/*z*): $[M + Na]^+$ calcd for $C_{36}H_{42}N_4NaO_6$, 649.2997; found, 649.2976.

Cyclization of 20 to pyrimidine-*N*-oxide 30:

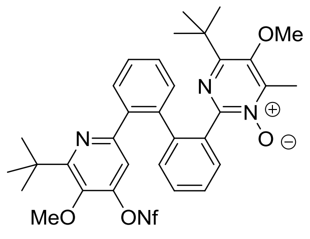

30

According to typical procedure 5, the reaction of enamide **20** (45 mg, 0.055 mmol) and NH_2OH•HCl (38 mg, 0.55 mmol) in MeOH (2 mL) afforded after purification by column chromatography (silica gel, EtOAc) pyrimidine-*N*-oxide **30** (24 mg, 54%) as a pale yellow oil.

4-*tert*-Butyl-2-{2'-[6-*tert*-butyl-5-methoxy-4-(nonafluorobutylsulfonyloxy)pyridin-2-yl]biphenyl-2-yl}-5-methoxy-6-methylpyrimidin-1-oxide (**30**): IR (ATR) ν: 3060−2870 (=C-H, C-H), 1550−1355 (C=C) cm^{-1}; ^{1}H NMR ($CDCl_3$, 500 MHz): δ 1.01, 1.35 (2 s, 9H each, *t*Bu), 2.44 (s, 3H, Me), 3.77, 3.91 (2 s, 3H each, OMe), 6.99 (s, 1H, Py), 7.01 (br d, *J* = 7.8 Hz, 1H, Ar), 7.11 (br d, *J* = 7.0 Hz, 1H, Ar), 7.18 (t, *J* = 7.4 Hz, 1H, Ar), 7.29−7.36 (m, 3H, Ar), 7.66 (brd, *J* = 7.4 Hz, 1H, Ar), 7.85 (dd, *J* = 7.8, 1.4 Hz, 1H, Ar) ppm; ^{13}C NMR ($CDCl_3$, 126 MHz): δ 12.5 (q, Me), 28.8, 29.4, 37.6, 38.8 (2 s, 2 q, *t*Bu), 61.7, 61.8 (2 q, OMe), 116.0 (d, Pyrid), 126.5, 127.4, 128.2, 129.4, 130.0, 130.2, 130.6, 131.4 (8 d, Ar), 132.5, 138.3, 140.0, 141.4, 145.6, 149.1, 149.2, 150.2 (8 s, Ar, Pyrid), 150.8, 152.7, 155.8, 163.6 (4 s, Pyrim) ppm; ESI–TOF (*m*/*z*): $[M + Na]^+$ calcd for $C_{36}H_{36}F_9N_3NaO_6S$, 832.2073; found, 832.2051.

Boekelheide rearrangement of 30:

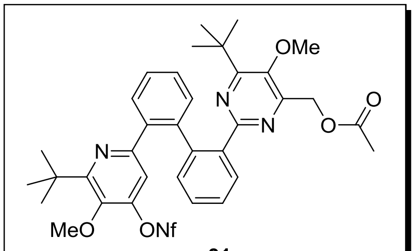

31

According to typical procedure 6, the reaction of pyrimidine-*N*-oxide **30** (24 mg, 0.029 mmol) and Ac_2O (0.05 mL, 0.53 mmol) in benzene (2 mL) provided after heating to 80 °C for 16 h and after purification by column chromatography (silica gel, hexanes/EtOAc = 9:1 to 4:1) compound **31** (14 mg, 55%) as a colorless viscous oil.

{6-*tert*-Butyl-2-[2'-(6-*tert*-butyl-5-methoxy-4-(nonafluorobutylsulfonyloxy)pyridin-2-yl)biphenyl-2-yl]-5-methoxypyrimidin-4-yl}methyl acetate (**31**): ^{1}H NMR ($CDCl_3$, 500 MHz): δ 0.98, 1.37 (2 s, 9H each, *t*Bu), 2.14 (s, 3H, Me), 3.78, 3.87 (2 s, 3H each, OMe), 5.14, 5.18 (AB system, J_{AB} = 13.1 Hz, 2H, OCH_2), 6.95 (dd, *J* = 7.7, 0.9 Hz, 1H, Ar), 6.99 (s, 1H, Py), 7.01 (dd, *J* = 7.5, 0.9 Hz, 1H, Ar), 7.19, 7.27 (2 dt, *J* = 7.5, 1.4 Hz, 1H each, Ar), 7.32 (dt, *J* = 7.7, 1.0 Hz, 1H, Ar), 7.34 (dt, *J* = 7.5, 1.0 Hz, 1H, Ar), 7.72, 7.90 (2 dd, *J* = 7.7, 1.0 Hz, 1H each, Ar) ppm; ^{13}C NMR ($CDCl_3$, 126 MHz): δ 20.2 (q, Me), 28.2, 28.8, 37.4, 38.2 (2 s, 2 q, *t*Bu), 61.1 (q, OMe), 61.3 (t, OCH_2), 61.9 (q, OMe), 115.4 (d, Pyrid), 126.2, 126.7, 127.5, 128.3, 129.5, 130.1, 130.2, 131.2 (8 d, Ar), 137.3, 137.5, 140.3, 141.2, 144.6, 148.5, 149.2, 152.7 (8 s, Ar, Pyrid), 155.7, 159.3, 162.8, 168.3 (4 s, Pyrim), 170.1 (s, C=O) ppm.

Oxidation of 23a with SeO_2 followed by reduction with $NaBH_4$:

Pyrimidine **23a** (0.145 g, 0.334 mmol) and SeO_2 (0.185 g, 1.67 mmol) were stirred in 1,4-dioxane (5 mL) at 90 °C for 3 d in an ACE-sealed tube. The resulting black metallic residue was filtered off using a small pad of celite and the filtrate was concentrated under reduced pressure. The crude product was purified by column chromatography (silica gel, hexanes/EtOAc = 15:1 to 3:1) to afford 0.120 g of a yellow oil, consisting of an inseparable mixture of the mono- and the dialdehyde.

The obtained mixture was dissolved in methanol (5 mL) and $NaBH_4$ (49 mg, 1.30 mmol) was added at 0 °C. Upon stirring the mixture was allowed to warm up to rt. After 2 h the reaction was quenched by the addition of ice and water (10 mL) and was diluted with Et_2O (20 mL). The layers were separated and the aqueous layer was extracted with Et_2O (2 × 20 mL). The combined organic layers were washed with brine (40 mL), dried

with Na_2SO_4 and filtered. The solvent was evaporated under reduced pressure and the obtained crude product was purified by column chromatography (silica gel, hexanes/EtOAc = 4:1 to 1:1) to afford diol **32a** [79 mg, 51% (over 2 steps)] and alcohol **32b** [37 mg, 25% (over 2 steps)], both as colorless viscous oils.

2,2'-(1,3-Phenylene)bis(6-*tert*-butyl-5-methoxypyrimidine-4,2-diyl)dimethanol (**32a**):

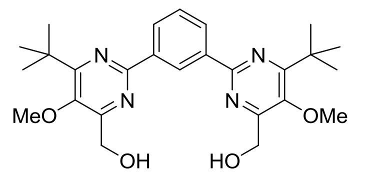

32a

IR (ATR) ν: 3450 (OH), 2965–2865 (=C-H, C-H), 1550–1370 (C=C) cm^{-1}; ^{1}H NMR ($CDCl_3$, 400 MHz): δ 1.50 (s, 18H, *t*Bu), 3.82 (s, 6H, OMe), 4.34 (t, *J* = 4.6 Hz, 2H, OH), 4.86 (d, *J* = 4.6 Hz, 4H, OCH_2), 7.58 (t, *J* = 7.8 Hz, 1H, Ar), 8.52 (dd, *J* = 7.8, 1.8 Hz, 2H, Ar), 9.59 (t, *J* = 1.8 Hz, 1H, Ar) ppm; ^{13}C NMR ($CDCl_3$, 101 MHz): δ 29.1, 38.5 (s, q, *t*Bu), 59.7 (q, OMe), 61.7 (t, OCH_2), 128.1, 128.8, 129.6 (3 d, Ar), 137.6 (s, Ar), 149.2, 156.8, 159.9, 168.7 (4 s, Pyr) ppm; ESI–TOF (*m*/*z*): $[M + Na]^+$ calcd for $C_{26}H_{34}N_4NaO_4$, 489.2472; found, 489.2492.

{6-*tert*-Butyl-2-[3-(4-*tert*-butyl-5-methoxy-6-methylpyrimidin-2-yl)phenyl]-5-methoxypyrimidin-4-yl}methanol (**32b**):

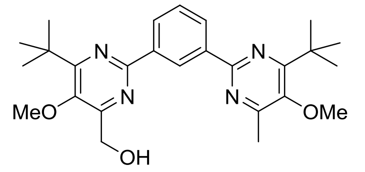

32b

IR (ATR) ν: 3445 (OH), 2955–2865 (=C-H, C-H), 1550–1375 (C=C) cm^{-1}; ^{1}H NMR ($CDCl_3$, 400 MHz): δ 1.48, 1.49 (2 s, 9H each, *t*Bu), 2.59 (s, 3H, Me), 3.81 (s, 6H, OMe), 4.40 (br s, 1H, OH), 4.85 (br s, 2H, OCH_2), 7.53–7.57 (m, 1H, Ar), 8.49 (t, *J* = 1.5 Hz, 1H, Ar), 8.51 (t, *J* = 1.5 Hz, 1H, Ar), 9.55 (t, *J* = 1.5 Hz, 1H, Ar) ppm; ^{13}C NMR ($CDCl_3$, 101 MHz): δ 20.0 (q, Me), 29.1, 29.2, 38.3, 38.5 (2 s, 2 q, *t*Bu), 59.7, 61.1, 61.7 (2 q, t, OMe, OCH_2), 127.9, 128.7, 129.2, 129.7 (4 d, Ar), 137.4, 138.5 (2 s, Ar), 149.1, 151.1, 156.9, 157.2, 159.8, 160.5, 167.9, 168.6 (8 s, Pyr) ppm; ESI–TOF (*m*/*z*): $[M + H]^+$ calcd for $C_{26}H_{35}N_4O_3$, 451.2704; found, 451.2708.

Allylation of 32a:

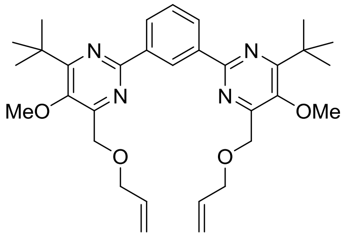

33

To a solution of diol **32a** (77 mg, 0.165 mmol) in dry THF (5 mL) was added NaH (40 mg, 1.7 mmol, 60% in mineral oil) at 0 °C. After 15 min stirring, tetraethylammonium iodide (4 mg, 0.02 mmol) and allyl bromide (208 mg, 1.72 mmol) were added at 0 °C. The reaction mixture was stirred over night while being allowed to warm up to rt. After dilution with Et_2O (10 mL), ice and water (5 mL) were slowly added. The layers were separated and the aqueous layer was extracted with Et_2O (2 × 20 mL). The combined organic layers were washed with brine, dried with Na_2SO_4 and filtered. The solvent was evaporated under reduced pressure and the crude product was purified by column chromatography (silica gel, hexanes/EtOAc = 9:1 to 4:1) to afford compound **33** (69 mg, 77%) as a colorless viscous oil.

1,3-Bis[4-(allyloxymethyl)-6-*tert*-butyl-5-methoxypyrimidin-2-yl)benzene (**33**): IR (ATR) ν: 3080–2865 (=C-H, C-H), 1550–1375 (C=C) cm^{-1}; ^{1}H NMR ($CDCl_3$, 400 MHz): δ 1.49 (s, 18H, *t*Bu), 3.92 (s, 6H, OMe), 4.27 (dt, *J* ≈ 5.7, 1.4 Hz, 4H, OCH_2), 4.72 (s, 4H, OCH_2), 5.24 (dq, *J* ≈ 10.4, 1.5 Hz, 2H, $=CH_2$), 5.37 (dq, *J* ≈ 17.2, 1.7 Hz, 2H, $=CH_2$), 5.96–6.06 (ddt, *J* = 17.2, 10.4, 5.7 Hz, 2H, CH=), 7.54 (t, *J* = 7.7 Hz, 1H, Ar), 8.51 (dd, *J* = 7.7, 1.8 Hz, 2H, Ar), 9.57 (t, *J* = 1.8 Hz, 1H, Ar) ppm; ^{13}C NMR ($CDCl_3$, 101 MHz): δ 29.2, 38.6 (q, s, *t*Bu), 63.2, 68.6, 72.2 (2 t, q, OCH_2, OMe), 117.7, 128.2, 128.6, 129.6, 134.6, 138.1 (t, 4 d, s, $CH=CH_2$, Ar), 151.4, 157.6, 158.4, 169.4 (4 s, Pyr) ppm; ESI–TOF (*m*/*z*): $[M + H]^+$ calcd for $C_{32}H_{43}N_4O_4$, 547.3279; found, 547.3291.

Ring-closing-metathesis of 33:

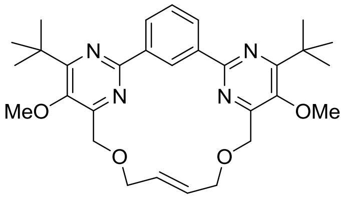

34

Diallyl ether **33** (65 mg, 0.119 mmol) was dissolved in CH_2Cl_2 (20 mL) and Grubbs-II-catalyst (11 mg, 0.013 mmol) was added. The solution was stirred at rt for 4 h under an argon atmosphere. The solvent was evaporated under reduced pressure and the residue was purified by column chromatography (silica gel, hexanes/EtOAc = 9:1) to afford macrocyclic compound **34** (45 mg, 73%) as a colorless oil.

Macrocyclic bispyrimidine 34: IR (ATR) ν: 2955–2840 (=C-H, C-H), 1535–1355 (C=C) cm^{-1}; ^{1}H NMR ($CDCl_3$, 400 MHz): δ 1.48 (s, 18H, *t*Bu), 3.88 (s, 6H, OMe), 4.37–4.38 (m, 4H, OCH_2), 4.82 (s, 4H, OCH_2), 6.47 (m_c, 2H, =CH), 7.54 (t, *J* = 7.8 Hz, 1H, Ar), 8.52 (dd, *J* = 7.8, 1.7 Hz, 2H, Ar), 9.86 (t, *J* = 1.7 Hz, 1H, Ar) ppm; ^{13}C NMR ($CDCl_3$, 101 MHz): δ 29.3, 38.5 (s, q, *t*Bu), 62.9, 67.1, 71.1 (2 t, q, OCH_2, OMe), 128.6, 128.9, 129.1, 130.5 (4 d, =CH, Ar), 138.0 (s, Ar), 150.7, 157.5, 159.1, 168.9 (4 s, Pyr) ppm; ESI–TOF (*m*/*z*): $[M + H]^+$ calcd for $C_{30}H_{38}N_4NaO_4$, 541.2785; found, 541.2812.

Dihydroxylation of 34:

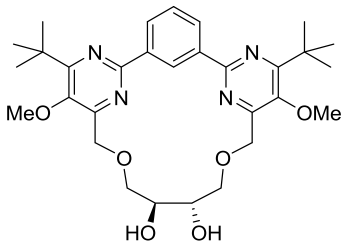

(*S*,*S*)-**35** [+ (*R*,*R*)-**35**]

Potassium osmate (4 mg, 0.01 mmol) and a 50% aq. solution of NMO (0.10 mL, 50 mg, 0.43 mmol) were added to a solution of compound **34** (45 mg, 0.087 mmol) in a mixture of acetone and water (6 mL, 5:1). The mixture was stirred at rt for 6 h. The reaction was quenched by adding Na_2SO_3. Acetone was removed under reduced pressure and the remaining aqueous phase was extracted with EtOAc (3 × 15 mL). The combined organic layers were washed with brine (40 mL), dried with Na_2SO_4, filtered and concentrated to dryness. The crude product was purified by column chromatography (silica gel, EtOAc) to furnish diol **35** (36 mg, 76%) as a colorless solid.

Macrocyclic bispyrimidine 35: mp 127–130 °C; IR (ATR) ν: 3350 (OH), 3005–2850 (=C-H, C-H), 1525–1460 (C=C) cm^{-1}; ^{1}H NMR ($CDCl_3$, 500 MHz): δ 1.49 (s, 18 H, *t*Bu), 3.81 (s, 6H, OMe), 3.86 (dd, *J* = 9.9, 7.1 Hz, 2H, OCH_2), 4.07 (dd, *J* = 9.9, 5.2 Hz, 2H, OCH_2), 4.28 (m_c, 2H, CH), 4.62 (br s, 2H, OH), 4.88, 4.97 (AB system, J_{AB} = 14.7 Hz, 4H, OCH_2), 7.57 (t, *J* = 7.7 Hz, 1H, Ar), 8.57 (dd, *J* = 7.7, 1.7 Hz, 2H, Ar), 9.58 (t, *J* = 1.7 Hz, 1H, Ar) ppm; ^{13}C NMR ($CDCl_3$, 126 MHz): δ 29.4, 38.6 (s, q, *t*Bu), 62.7, 68.0, 70.0, 73.8 (q, 2 t, d, OMe, OCH_2, COH), 127.9, 128.7, 130.0 (3 d, Ar), 137.9 (s, Ar), 150.3, 157.8, 159.7, 169.3 (4 s, Pyr) ppm; ESI–TOF (*m*/*z*): $[M + H]^+$ calcd for $C_{30}H_{41}N_4O_6$, 553.3021; found, 553.3042.

Esterification of 35 with (*S*)-MTPA-Cl:

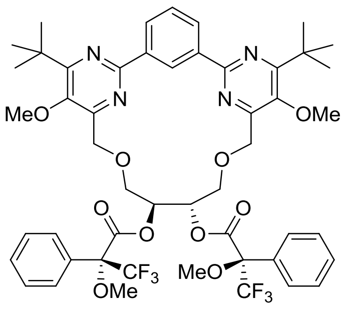

(*R*,*S*,*S*,*R*)-**36** [+ (*R*,*R*,*R*,*R*)-**36**]

Diol **35** (35 mg, 63 µmol), NEt_3 (40 µl, 288 µmol) and DMAP (one crystal) were dissolved in CH_2Cl_2 (1.0 mL). (*S*)-3,3,3-trifluoro-2-methoxy-2-phenylpropanoyl chloride (60 µl, 321 µmol) was added dropwise and the reaction was stirred for 2 d at rt. Since TLC analysis indicated incomplete conversion additional DMAP (two crystals), NEt_3 (40 µl, 288 µmol) and (*S*)-3,3,3-trifluoro-2-methoxy-2-phenylpropanoyl chloride (60 µl, 321 µmol) were added and stirring at rt was continued for 3 d. The reaction mixture was diluted with CH_2Cl_2 (2 mL) and was filtered through silica gel (CH_2Cl_2/EtOAc = 20:1). After evaporation of the solvent the obtained crude product was purified by column chromatography (silica gel, hexanes/EtOAc = 10:1) to furnish the bis-(*R*)-Mosher ester **36** (55 mg, 89%) as a colorless oil.

Bis-(*R*)-MTPA ester 36: IR (ATR) ν: 3065–2855 (=C-H, C-H), 1750 (C=O), 1550, 1450 (C=N, C=C), 1385–1365, 1265–1245, 1165 cm^{-1}; ^1H NMR ($CDCl_3$, 700 MHz): δ 1.49, 1.50 (2 s, 18 H each, *t*Bu), 3.46, 3.50, 3.76, 3.82 (4 s, 6H each, OMe), 4.15 (dd, J = 11.1, 3.0 Hz, 2H, OCH_2), 4.27 (dd, J = 11.0, 8.3 Hz, 2H, OCH_2), 4.28–4.30 (m, 4H, OCH_2), 4.58, 4.63 (AB-system, 4H, J_{AB} = 14.5 Hz, OCH_2Py), 4.70, 4.74 (AB system, J_{AB} = 14.0 Hz, 4H, OCH_2Py), 5.88–5.90 (m, 2H, OCH), 5.93 (m_c, 2H, OCH), 7.28–7.30, 7.33–7.40 (2 m, 4H, 8H Ph), 7.54–7.60 (m, 10H, Ph, Ar), 8.54 (dd, J = 7.7, 1.8 Hz, 2H, Ar), 8.56 (dd, J = 7.7, 1.7 Hz, 2H, Ar), 9.36, 9.43 (2 m_c, 1H each, Ar) ppm; an unambiguous assignement of the signals to the individual diastereomers was not possible; ^{13}C NMR ($CDCl_3$, 176 MHz): δ 29.1$^{\neq}$, 38.45, 38.48 (s, 2 q, *t*Bu), 55.3, 55.5, 62.8, 63.0 (4 q, OMe), 67.5, 67.9 (2 t, OCH_2Py), 68.9, 69.8, (2 t, OCH_2), 72.3, 72.9 (2 d, OCH), 84.6 (q, $^2J_{CF}$ = 28.0 Hz, CCF_3), 84.9 (q, $^2J_{CF}$ = 27.7 Hz, CCF_3), 123.2$^{¥}$ (q, $^1J_{CF}$ = 288.3 Hz, CF_3), 127.4, 127.7 (2 d, Ph), 127.9, 128.1 (2 d, Ar), 128.2, 128.3 (2 d, Ph), 128.6, 128.7 (2 d, Ar), 129.39, 129.49, 129.54, 129.61 (4 d, Ph, Ar), 131.6, 132.0 (2 s, Ph), 137.78, 137.83 (2 s, Ar), 150.2, 150.4, 157.27, 157.33, 158.5, 158.8 (6 s, Py), 165.7, 166.0 (2 s, C=O), 169.1, 169.2 (2 s, Py) ppm; $^{\neq}$ very strong intensity; $^{¥}$ unambiguous assignment of a seccond signal was not possible;

^{19}F NMR ($CDCl_3$, 376 MHz): δ –71.7, –71.5 (2 s, 3 F each, CF_3) ppm; ESI–TOF (*m*/*z*): $[M + Na]^+$ calcd for $C_{50}H_{54}F_6N_4NaO_{10}$, 1007.3636; found, 1007.3647.

Copies of ^{1}H, ^{13}C and ^{19}F NMR spectra (representative examples)

^{1}H and ^{13}C NMR of compound 14:

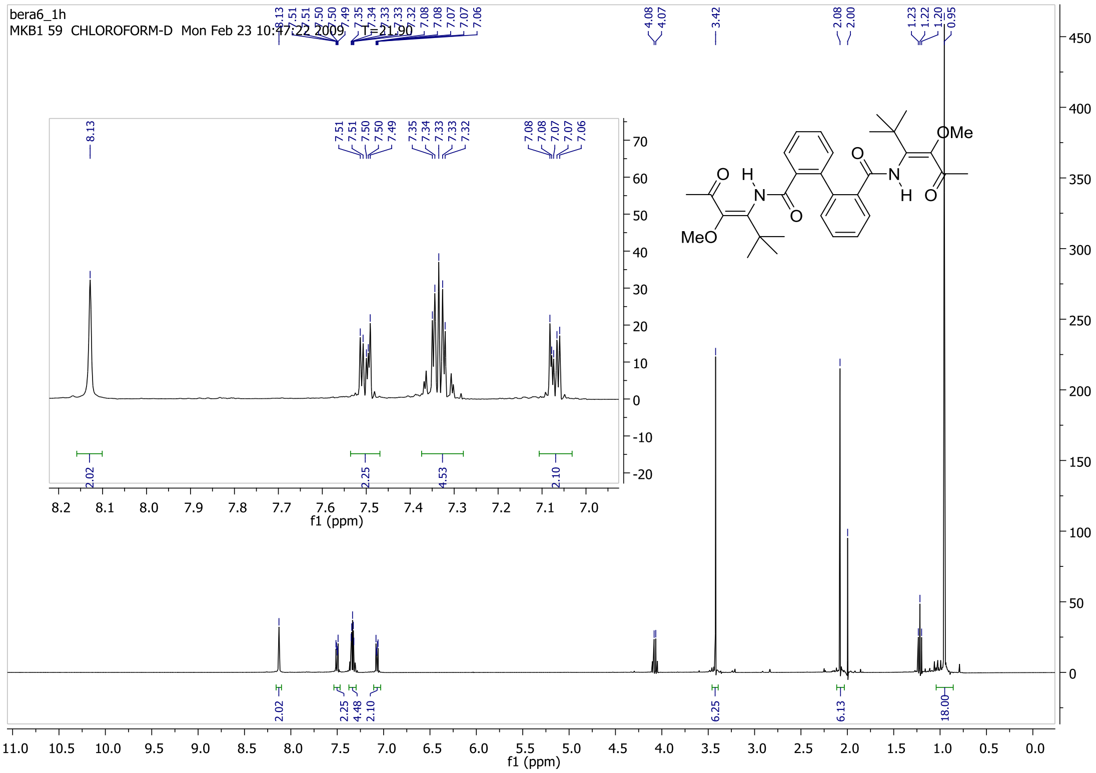

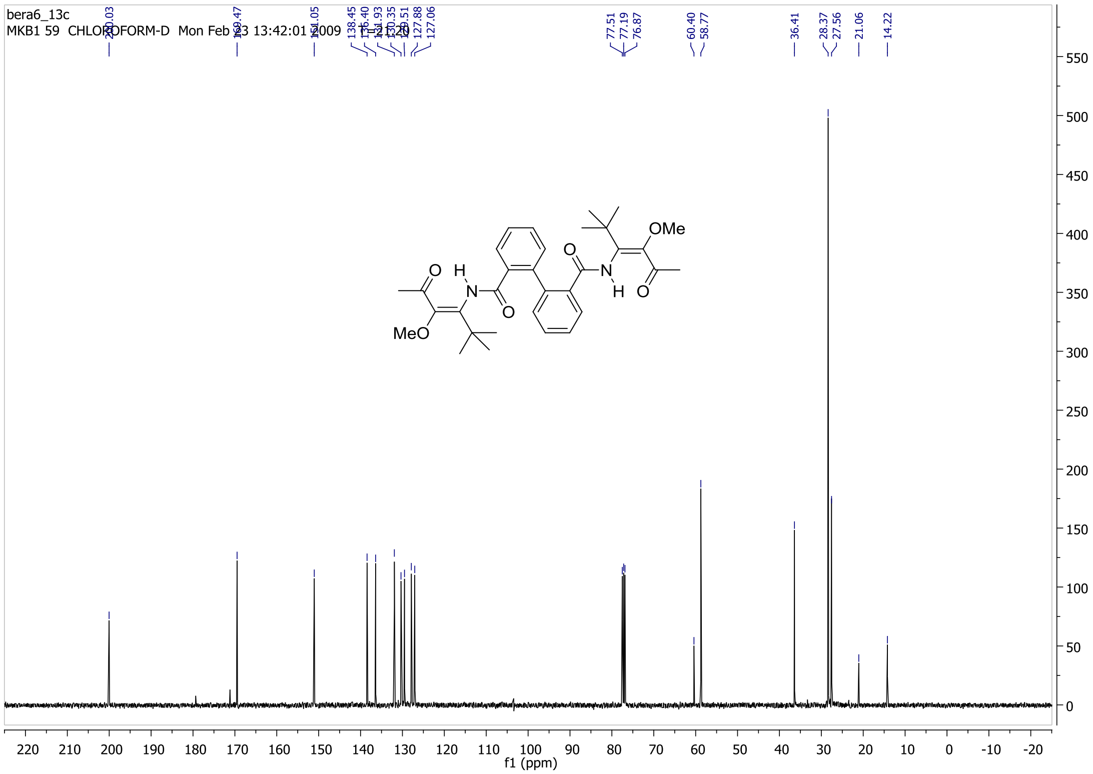

^{1}H and ^{13}C NMR of compound 17:

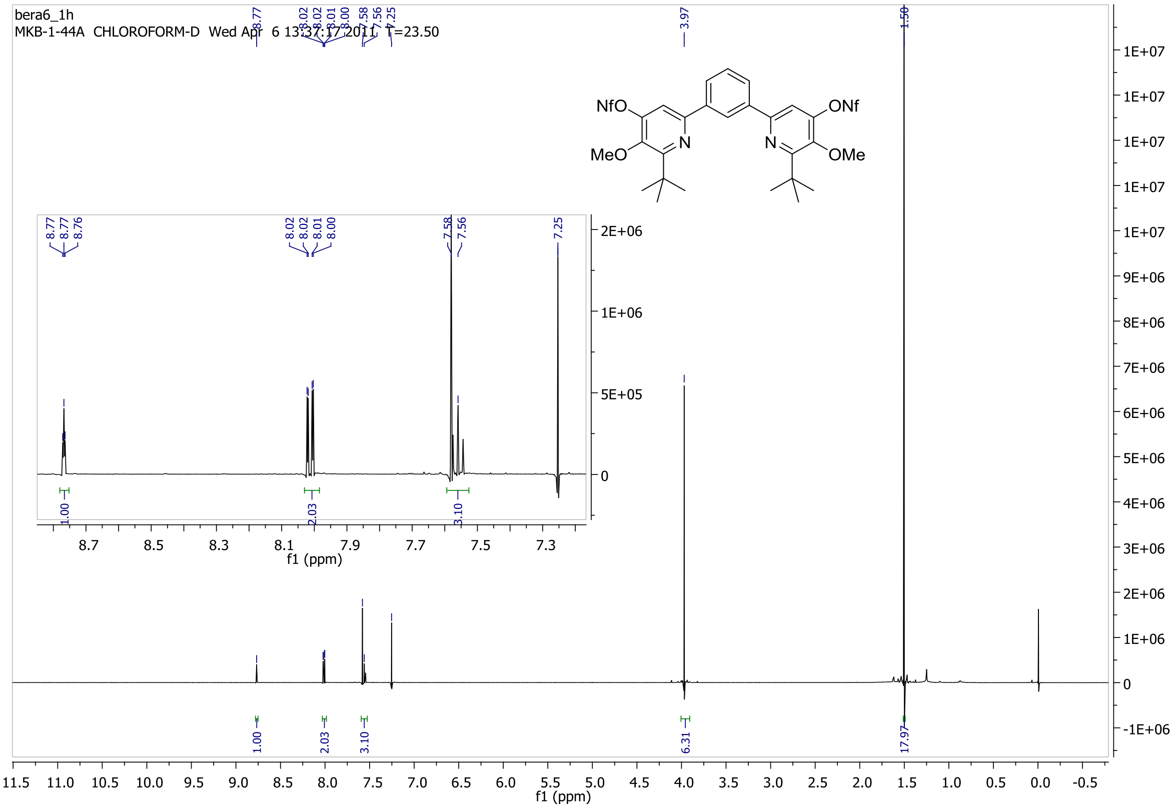

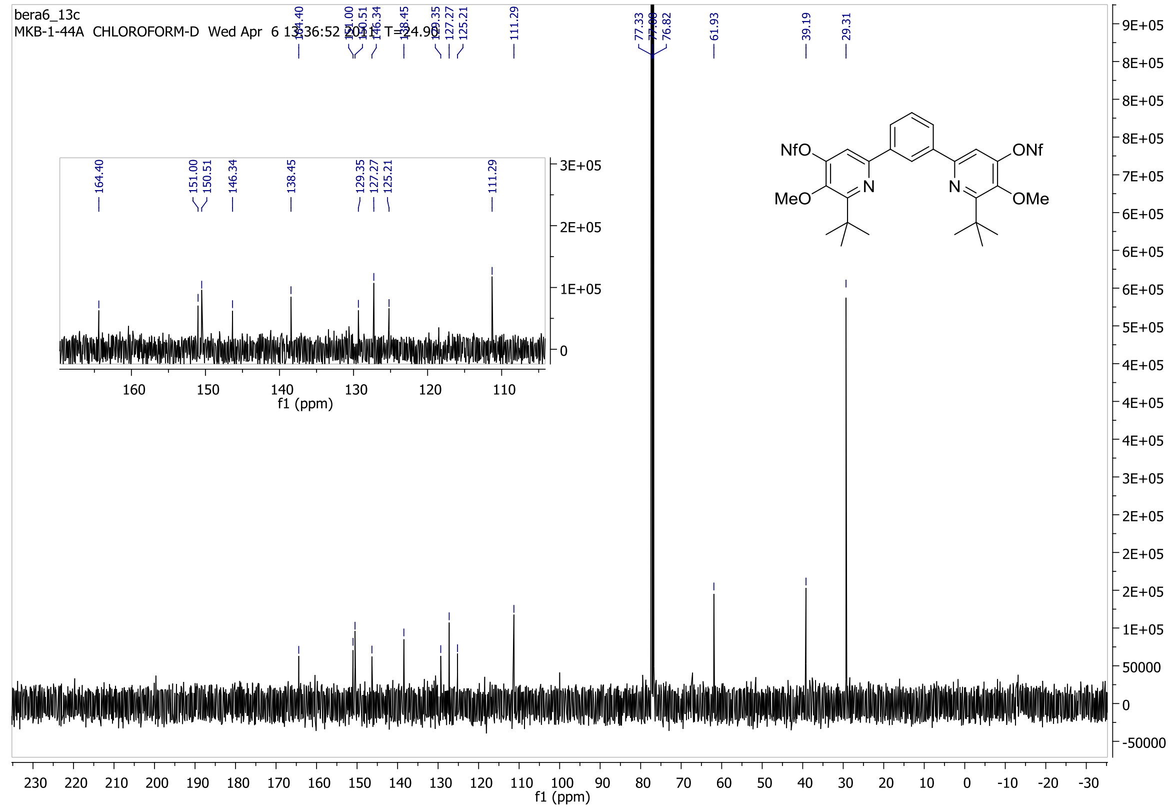

^{19}F NMR of compound 17:

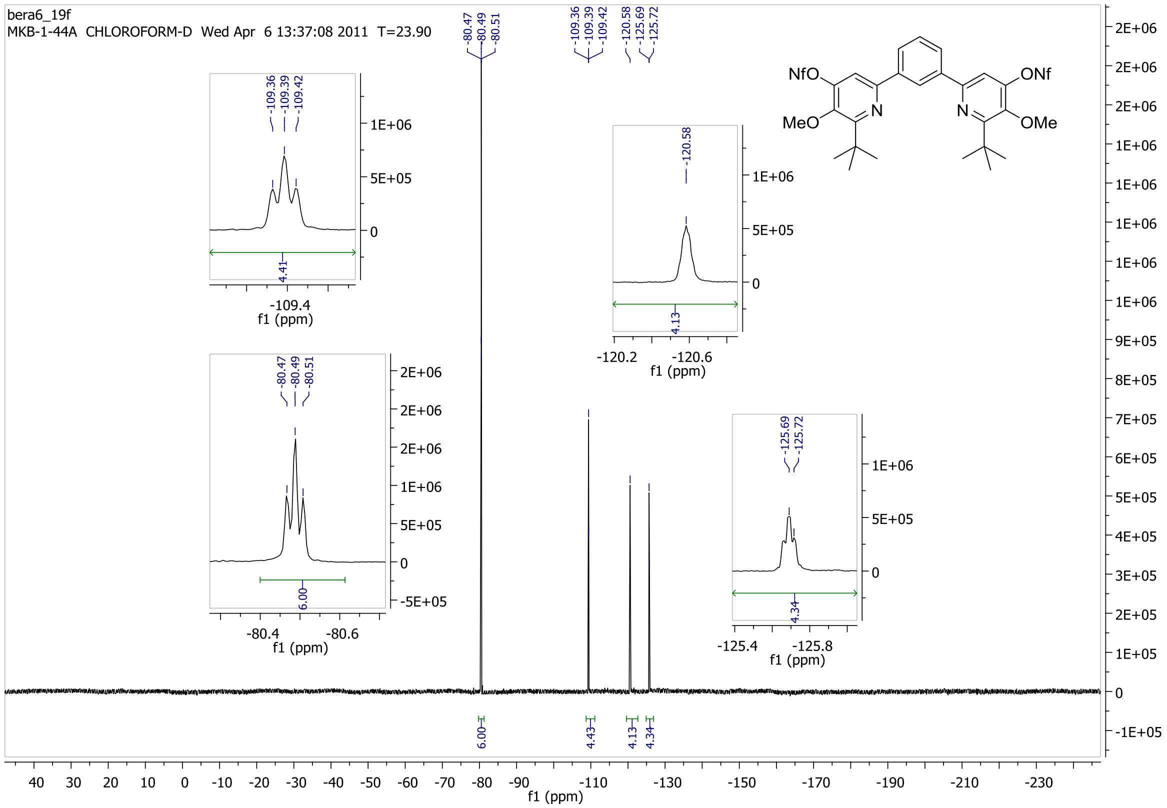

¹H and ¹³C NMR of compound 25:

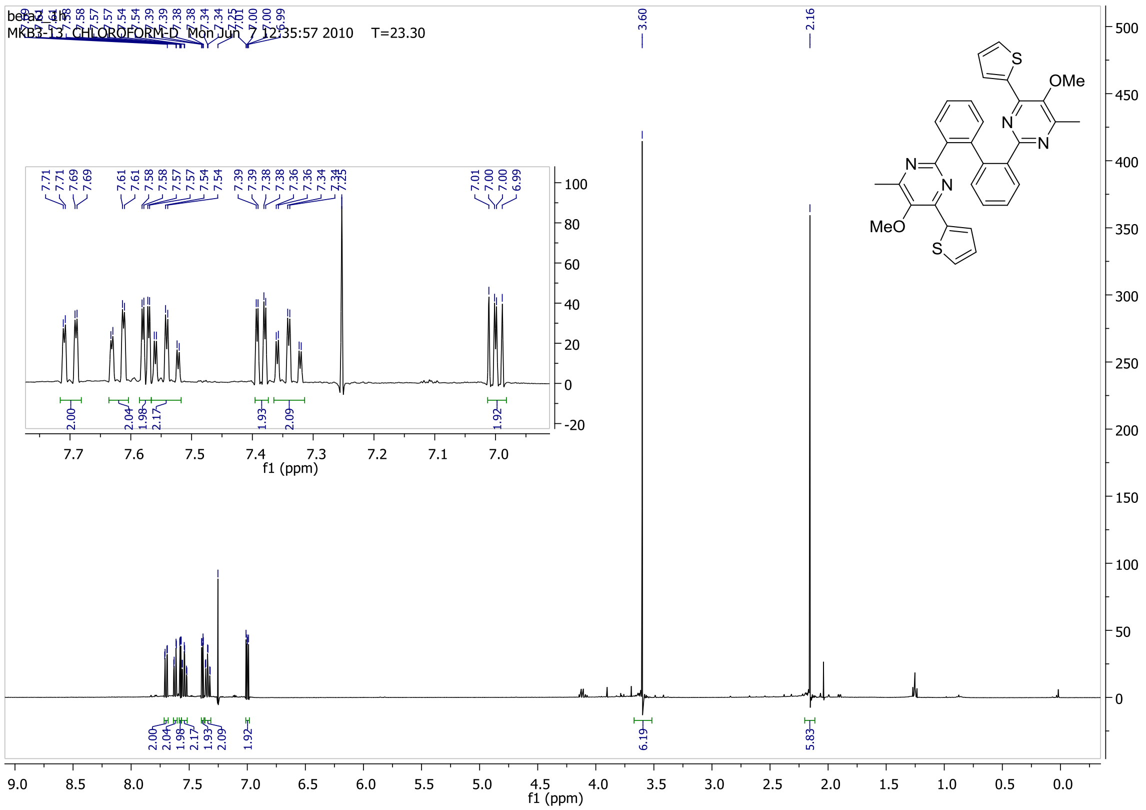

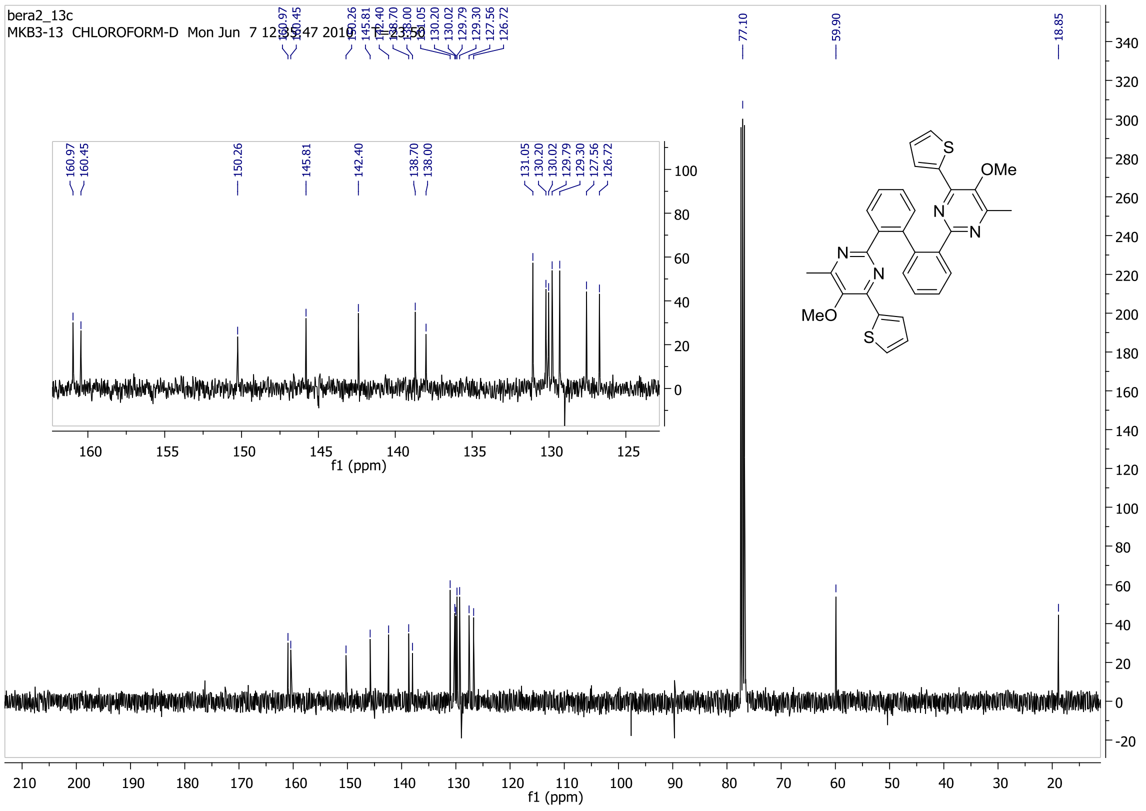

^{1}H and ^{13}C NMR of compound 26:

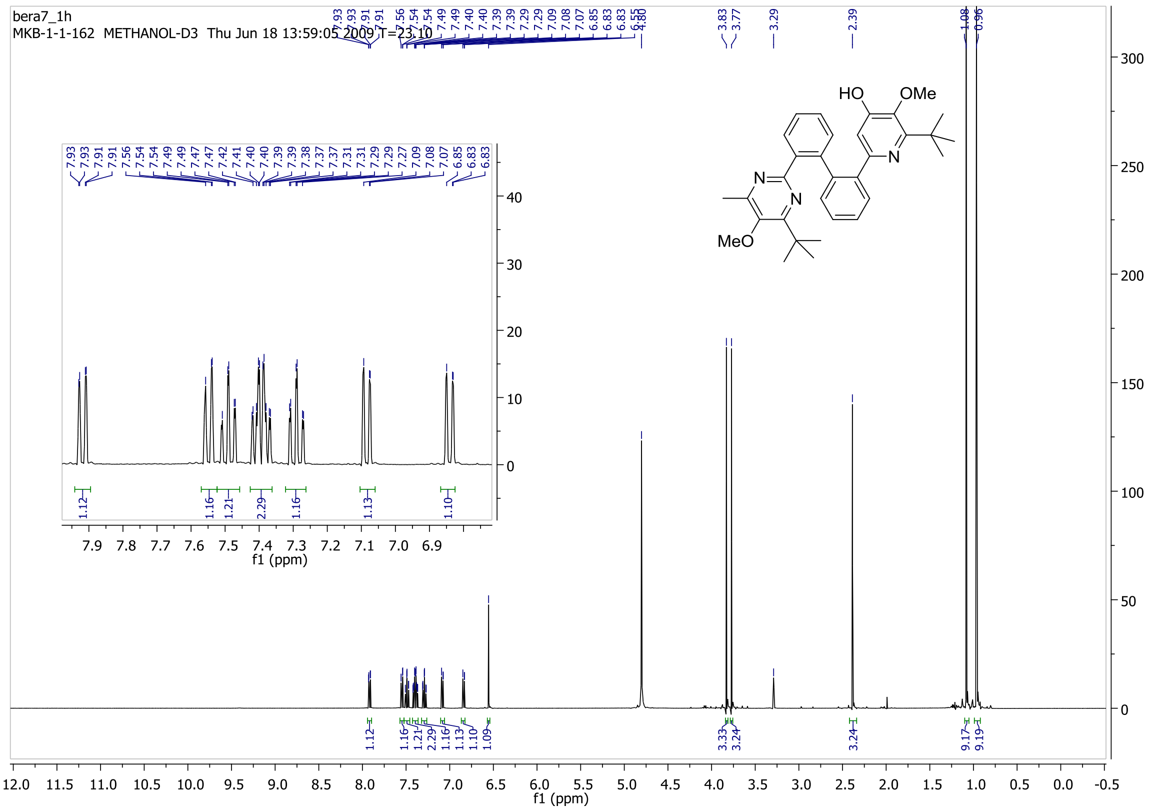

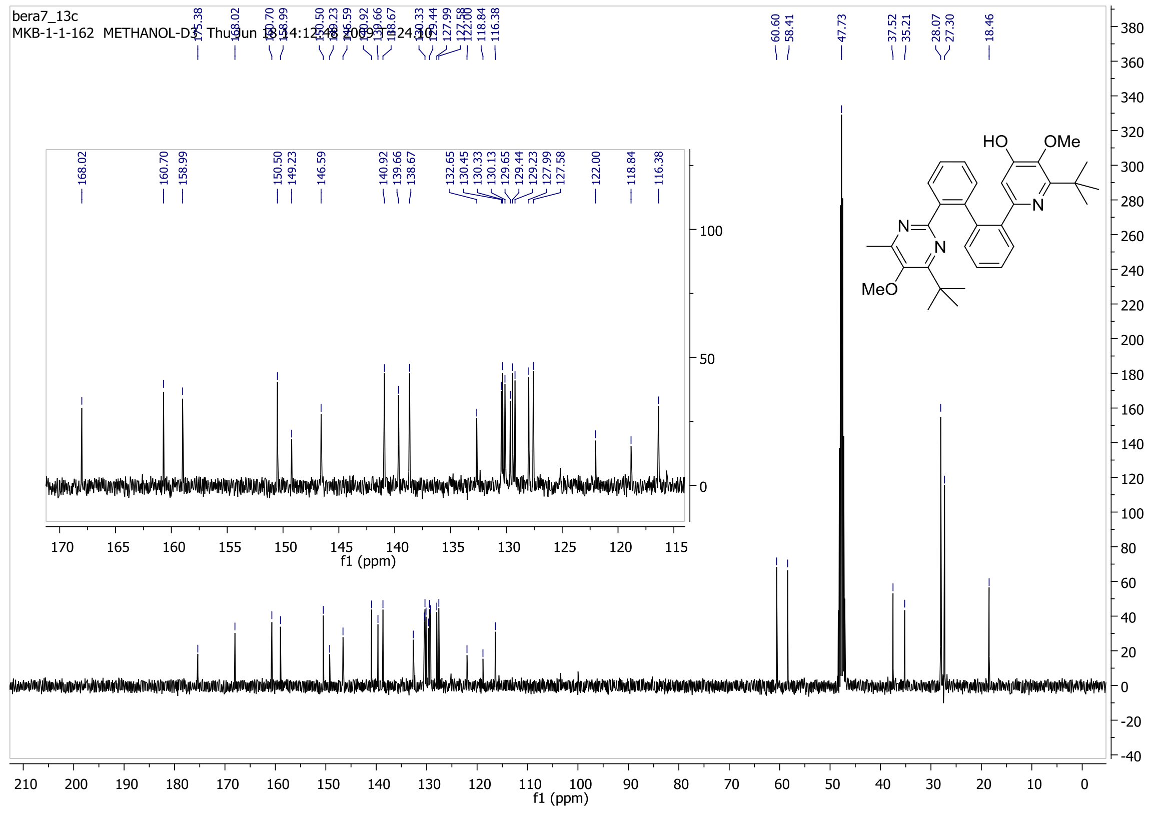

^{1}H and ^{13}C NMR of compound 30:

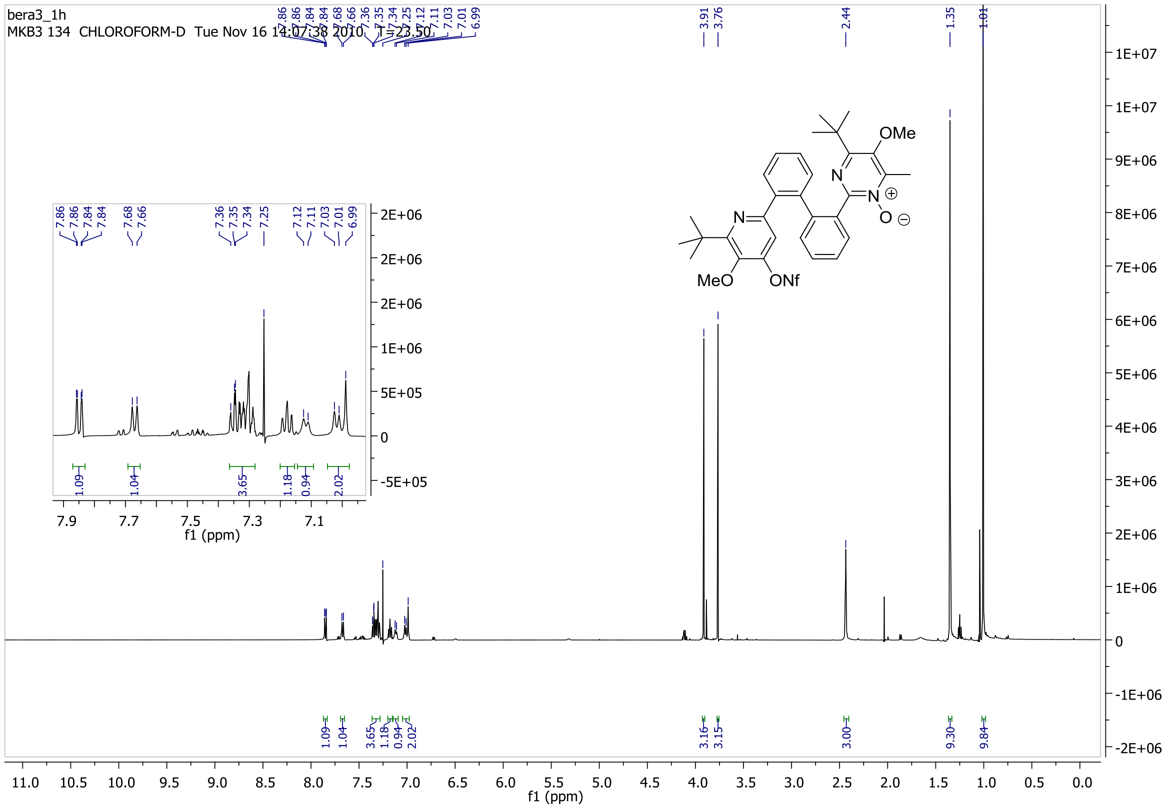

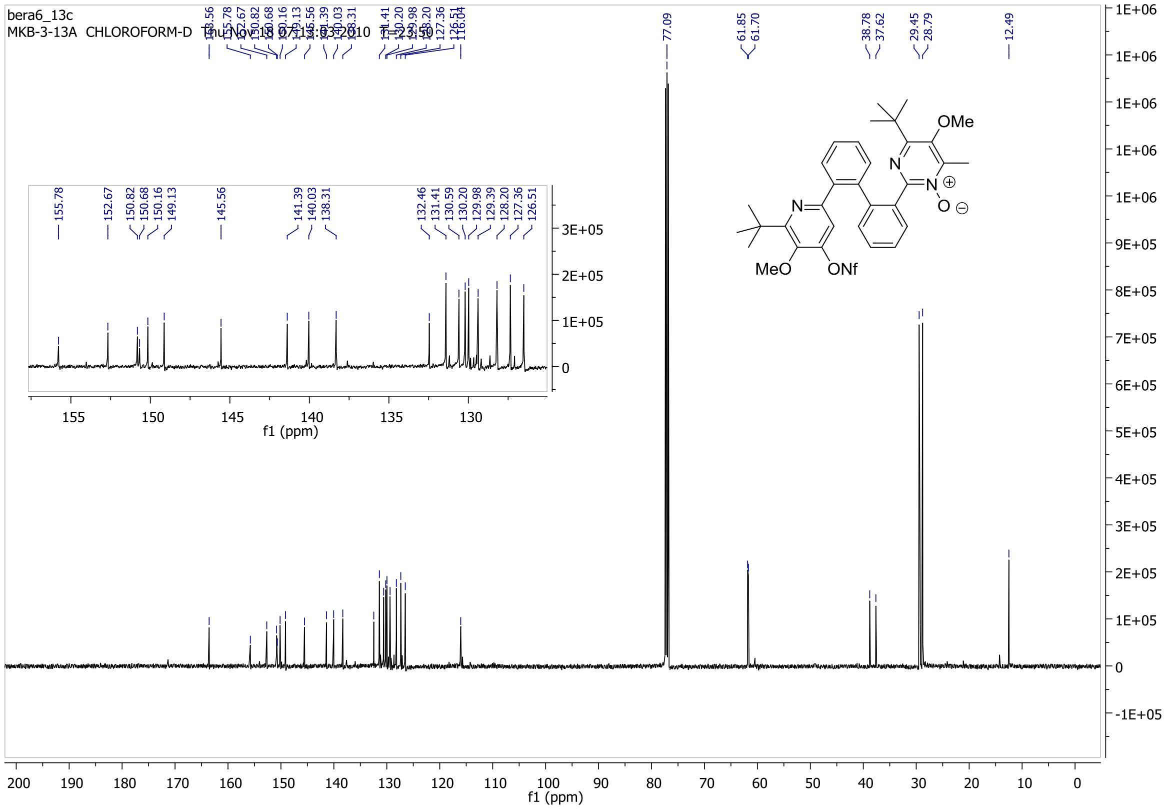

^{1}H and ^{13}C NMR of compound 31:

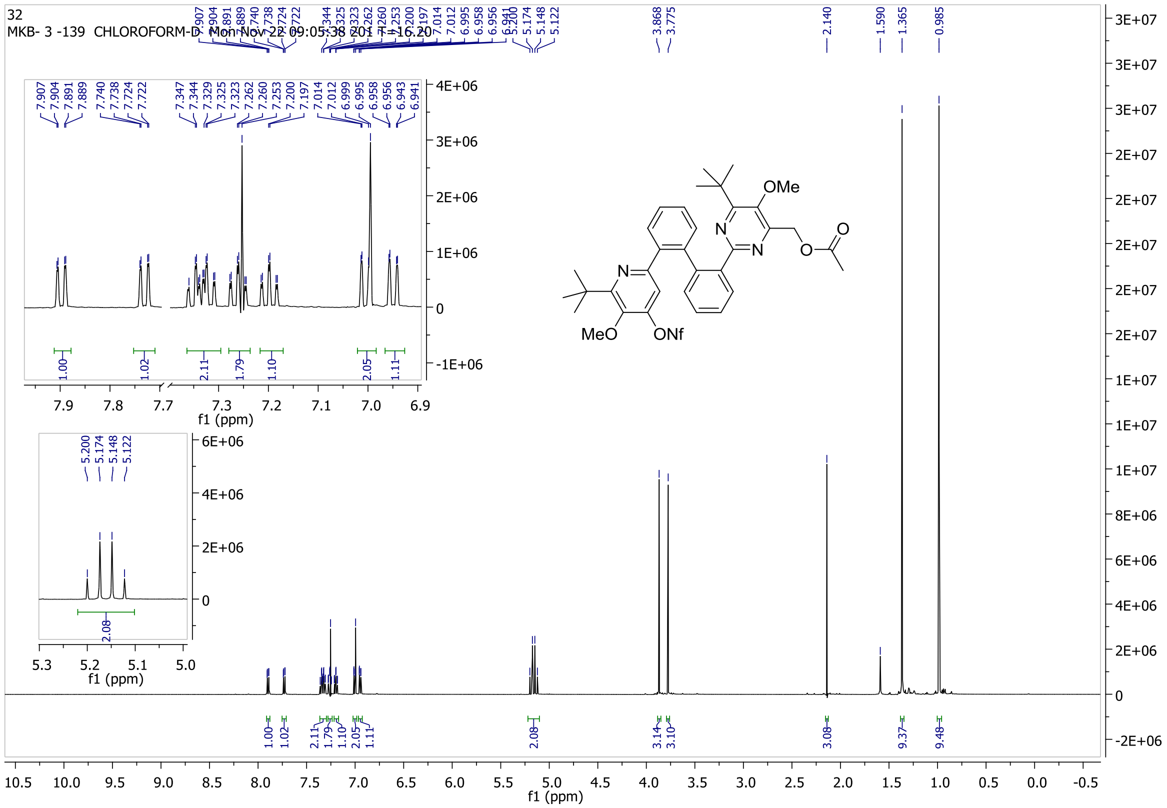

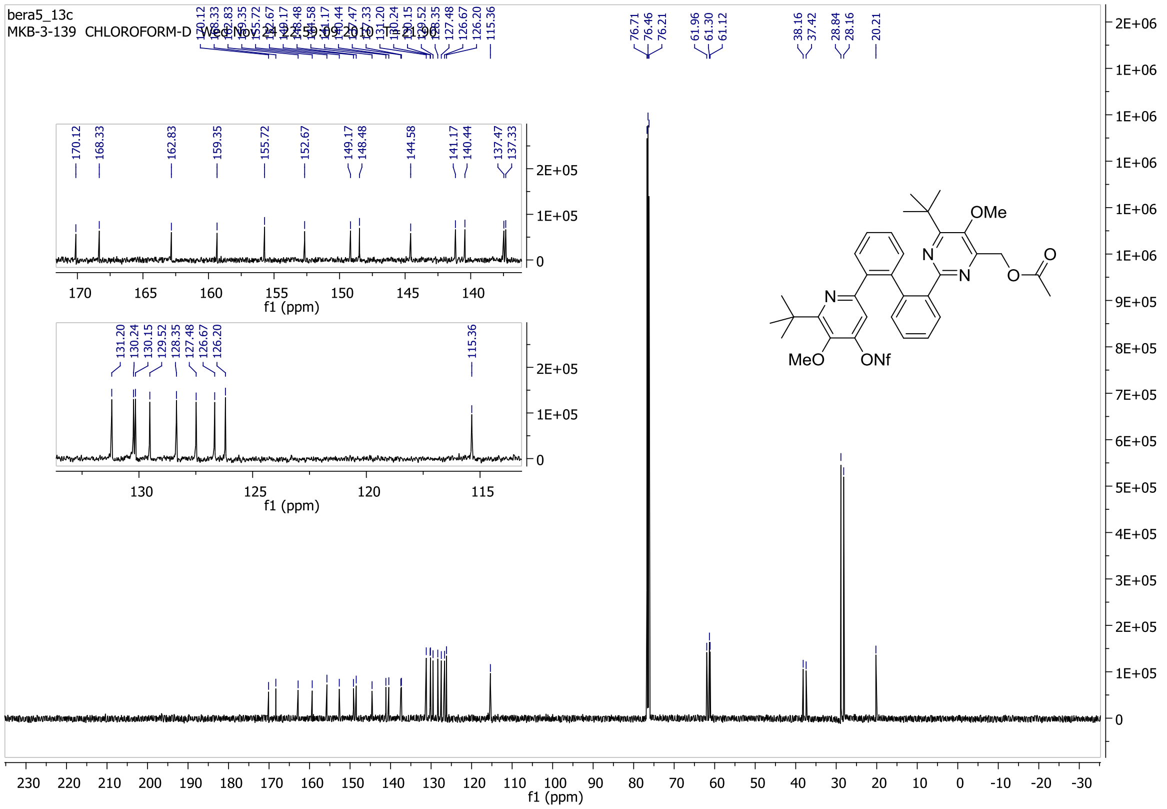

^{1}H and ^{13}C NMR of compound 33:

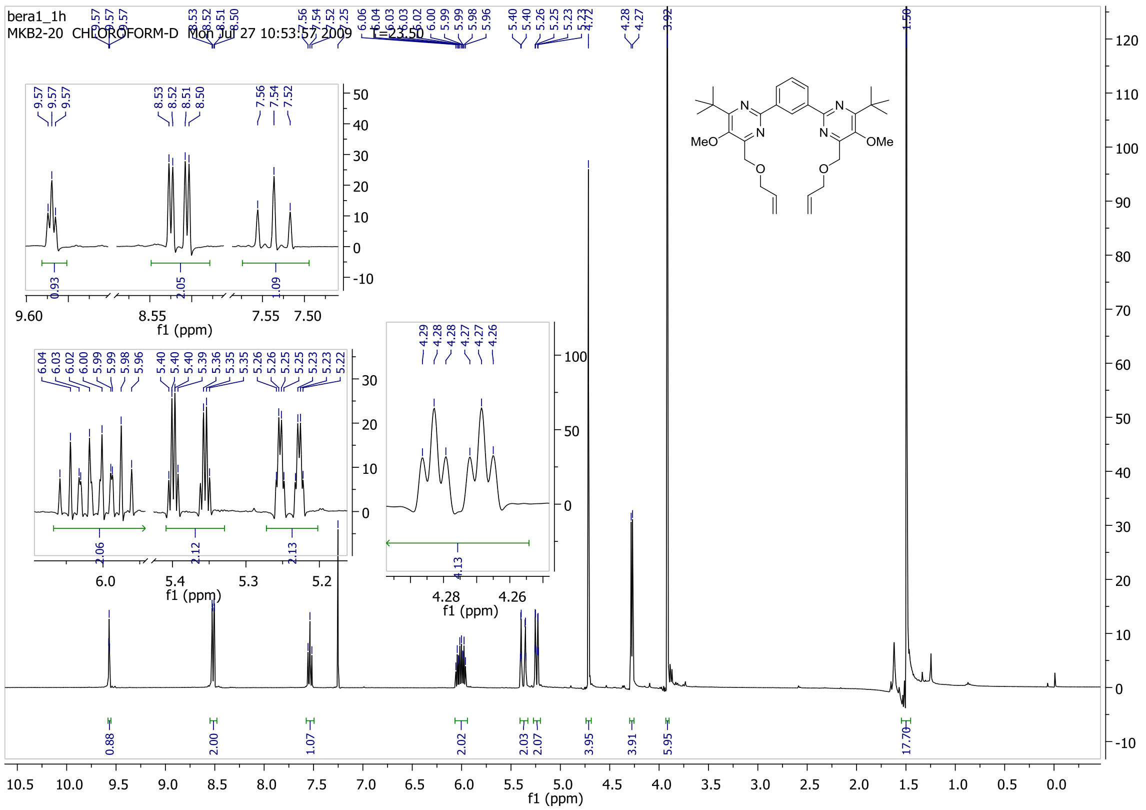

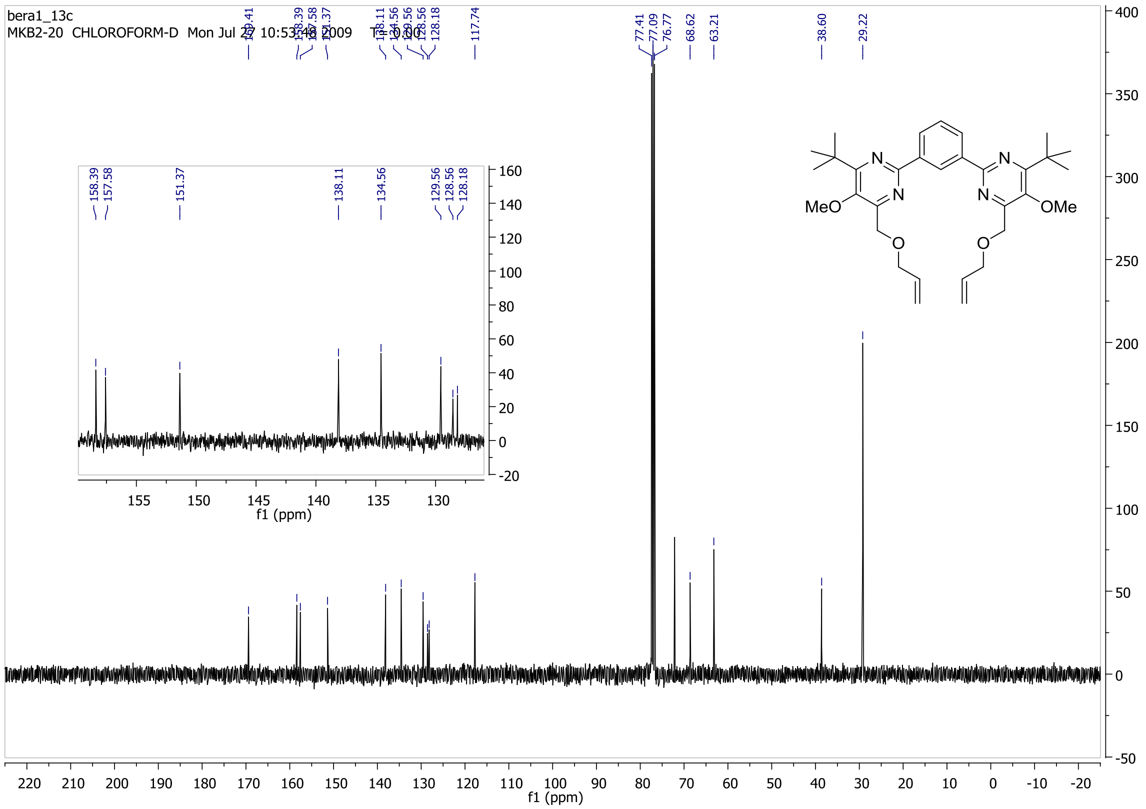

^{1}H and ^{13}C NMR of compound 34:

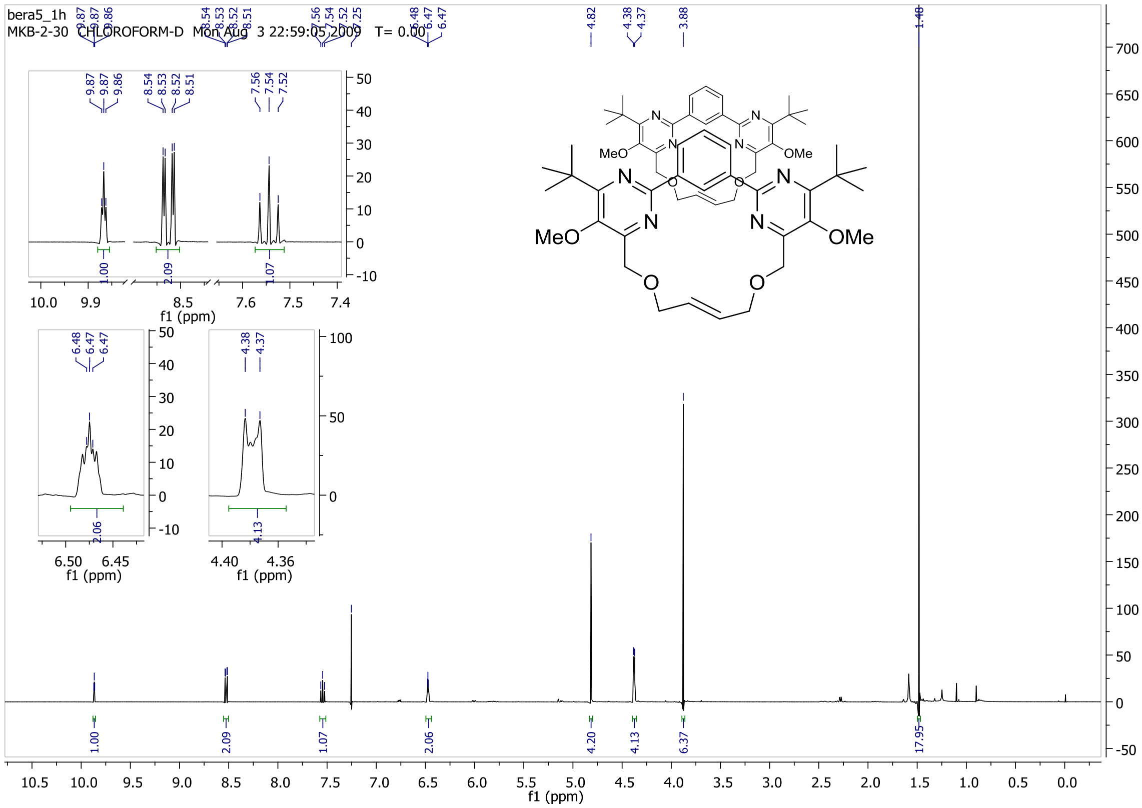

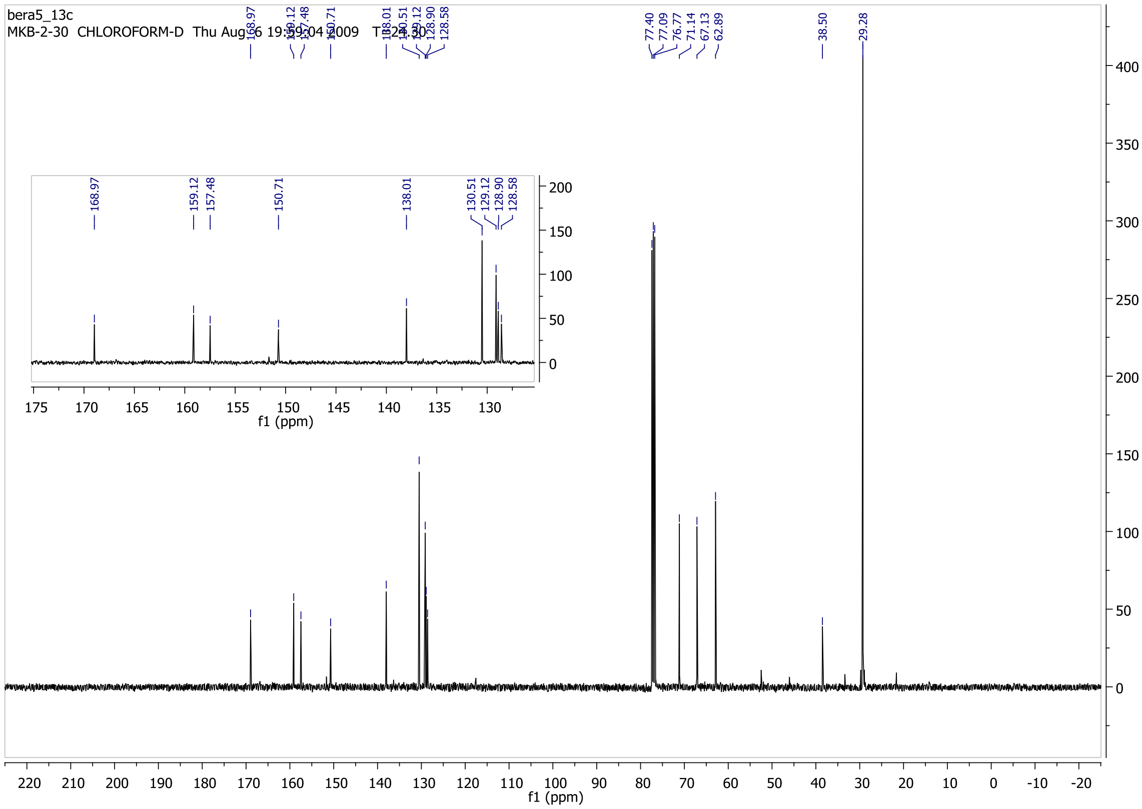

^1H and ^{13}C NMR of compound 36:

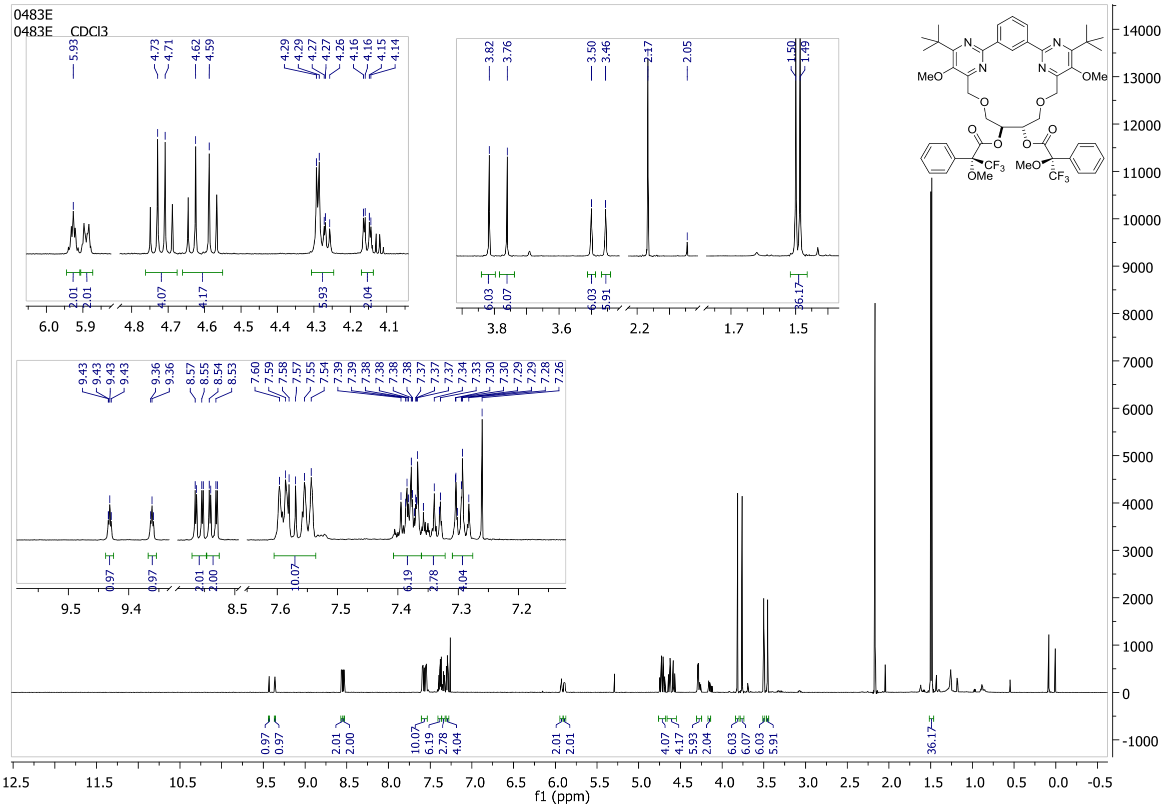

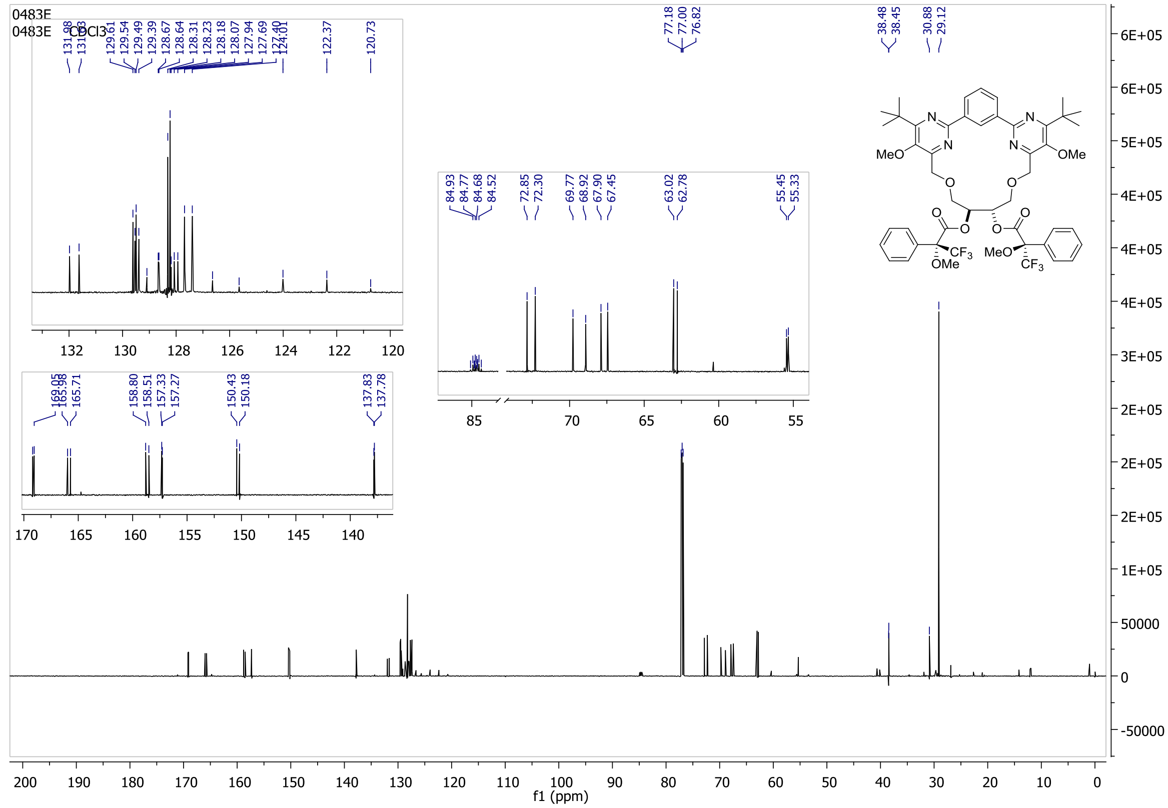

^{19}F NMR of compound 36:

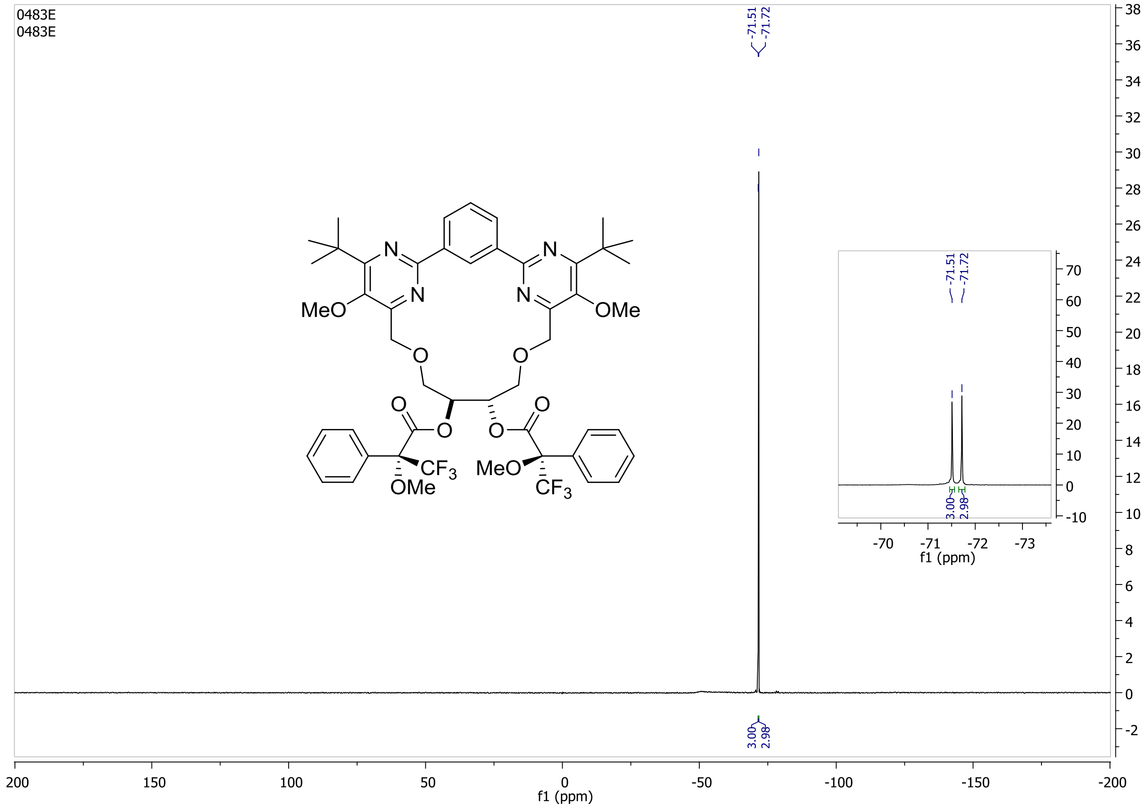

UV–vis and emission spectra of compound 22:

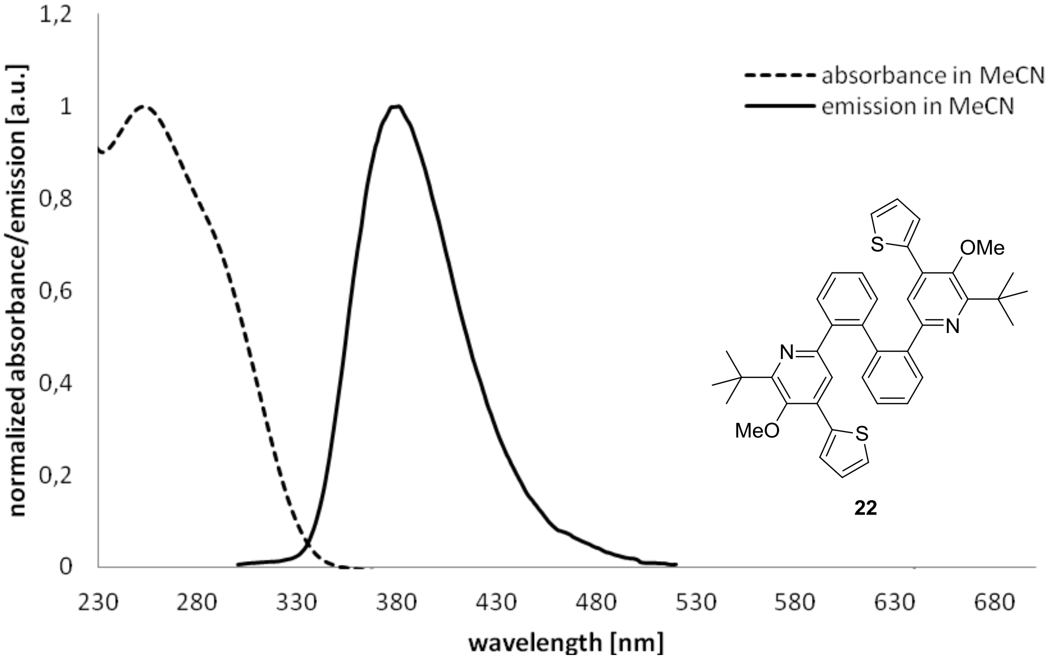

Absorbance and emission (after excitation at λ = 253 nm) were recorded at c = 1.08 × 10^{-5} mol/L in 1 cm quartz cuvettes.
